# Directed Structural Evolution of Nickel Nanoparticles into Atomically Dispersed Sites for Efficient CO_2_ Electroreduction.

**DOI:** 10.1002/smll.202505521

**Published:** 2025-09-05

**Authors:** Xiao Li, Tao Gan, Xinhua Gao, Bing Li, Juan Peng, Yang Ji, Shenghua Chen, Jian Zhang, Junjun Zhang, Pradip Kumar Das, Vinoth Ramalingam, Maolin Zhang, Pengfei Zhang, Karthik Peramaiah, Yajun Qiu

**Affiliations:** ^1^ State Key Laboratory of High‐efficiency Utilization of Coal and Green Chemical Engineering College of Chemistry and Chemical Engineering Ningxia University Yinchuan Ningxia 750021 P. R. China; ^2^ Shanghai Synchrotron Radiation Facility Shanghai Advanced Research Institute Chinese Academy of Sciences Shanghai 201204 P. R. China; ^3^ School of Chemistry National Innovation Platform (Center) for Industry‐Education Integration of Energy Storage Technology Xi'an Jiaotong University Xi'an 710049 P. R. China; ^4^ Key Laboratory of Carbon Materials of Zhejiang Province Key Lab of Biohealth Materials and Chemistry of Wenzhou College of Chemistry and Materials Engineering Wenzhou University Wenzhou Zhejiang 325035 P. R. China; ^5^ Institute of Institute of Sustainability for Chemicals Energy, and Environment (ISCE2) Agency for Science, Technology and Research (A*STAR) 1 Pesek Road Singapore 627833 Singapore; ^6^ School of Computing Engineering and Technology Robert Gordon University Garthdee Road Aberdeen AB10 7GJ UK; ^7^ Institute of Environment and Sustainable Development in Agriculture Chinese Academy of Agricultural Sciences Beijing 100081 P. R. China; ^8^ School of Chemistry and Chemical Engineering Shanghai Jiao Tong University Shanghai 200240 P. R. China

**Keywords:** activation, CO_2_ electroreduction, improved active sites density, Ni single‐atom, structural evolution

## Abstract

Electrochemical CO_2_ reduction (CO_2_RR) to carbon monoxide (CO) offers a sustainable pathway for carbon utilization, yet challenges remain in terms of improving selectivity and activity. Herein, we report a Ni/NC catalyst synthesized via a milling ‐ pyrolysis method, in which Ni particles anchored on nitrogen‐doped carbon (NC) are electrochemically activated under an Ar atmosphere, leading to their structural evolution into single‐atom Ni sites. After activation in Ar atmosphere, the current density nearly doubles (from ≈30 to ≈60 mA cm^−2^), and concurrently, the Faradaic efficiency of CO stays at ∼90% with the potential set to ‐0.8 V vs. RHE. Comprehensive characterizations, including X‐ray photoelectron spectroscopy (XPS), aberration ‐ corrected scanning transmission electron microscopy (AC ‐ STEM), along with extended X ‐ ray absorption fine structure (EXAFS), confirm the change of Ni particles into atomically dispersed Ni‐N_x_ moieties during activation. Notably, in situ Raman spectroscopy identifies ^*^COOH as the key intermediate, while electrochemical analyses reveal accelerated charge transfer and favorable kinetics for Ar‐Ni/NC. Additionally, the catalyst shows great selectivity and stability over 24 hours of non ‐ stop operation. This study emphasizes the dynamic change of Ni active sites under working conditions, offering useful ideas for designing transition metal catalysts for large ‐ scale CO_2_ to CO conversion.

## Graphical abstract

A Ni/NC catalyst synthesized via a milling‐pyrolysis method undergoes electrochemical activation in Ar, transforming Ni particles into atomically dispersed Ni‐N_x_ sites. Advanced characterizations confirm this structural evolution. The activated catalyst exhibits enhanced activity and stability, offering key insights into designing efficient, low‐cost transition metal catalysts for electrochemical CO_2_ reduction to carbon monoxide.

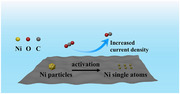

## 1 Introduction

As a strategy for promoting a circular carbon economy, the electrochemical CO_2_ reduction reaction (CO_2_RR) enables the efficient transformation of CO_2_ into high ‐ value chemicals and fuels, thereby promoting sustainable carbon utilization.^[^ ^1^, ^2^, ^3^, ^4^, ^5^, ^6^, ^7^ ^]^ Extensive research has been dedicated to converting CO_2_ into various C_1_ and C_2_ value‐added products. Among these, carbon monoxide (CO) stands out as the most viable product owing to its straightforward two ‐ electron transfer process.^[^ ^8^ ^]^ Besides, CO serves as a crucial intermediate in the synthesis of chemical raw materials via Fischer‐Tropsch chemistry, enabling the cascade conversion of CO_2_ into higher‐value C_2__+_ products (CO_2_→CO→C_2__+_).^[^ ^9^, ^10^, ^11^, ^12^, ^13^ ^]^ So far, catalysts based on noble metals like Au and Ag display excellent selectivity for CO production under electrochemical conditions.^[^ ^14^, ^15^ ^]^ However, their high cost and limited current density pose significant challenges for practical utilization. Thus, creating economical catalysts with strong activity and selectivity is essential to achieve large ‐ scale CO_2_ to CO conversion.^[^ ^16^, ^17^, ^18^ ^]^

Previous studies have shown that atomically dispersed 3d‐transition metal sites anchored to nitrogen‐doped carbon (M‐NC) materials are among the most effective electrocatalysts for the reduction of CO_2_ to CO with high selectivity and activity.^[^ ^1^, ^19^, ^20^, ^21^ ^]^ These catalysts not only exhibit high activity but also effectively inhibit the competitive hydrogen evolution reaction (HER). As a consequence, considerable work has been devoted to developing 3d transition metal ‐ based M–N–C catalysts for CO_2_ reduction. Among these, nickel‐based (Ni–N–C) catalysts have garnered particular attention due to their excellent catalytic performance.^[^ ^22^, ^23^, ^24^, ^25^, ^26^, ^27^ ^]^ The excellent catalytic performance of Ni–N–C materials is ascribed to their distinctive electronic structure, which arises from the interaction between Ni, nitrogen, and the carbon matrix, which facilitates efficient CO_2_ adsorption and activation.^[^ ^28^, ^29^, ^30^, ^31^, ^32^, ^33^, ^34^, ^35^ ^]^ Additionally, the carbon support enhances structural stability by preventing Ni agglomeration, while the atomically dispersed Ni sites ensure maximal utilization of active centers, thereby boosting catalytic efficiency.^[^ ^36^, ^37^, ^38^ ^]^ Despite their structural advantages, conventional Ni single‐atom catalysts prepared via traditional pyrolysis and acid‐leaching methods are often accompanied by Ni nanoparticles, which are considered electronic regulators that enhance the catalytic activity of Ni–N_x_ sites and are typically regarded as stable due to their encapsulating carbon layers.^[^ ^39^, ^40^, ^41^ ^]^ However, the long‐term stability of Ni particles in electrolysis is controversial. It has been demonstrated that environmental factors and reactive intermediates can cause structural changes during electrochemical reactions, resulting in the formation of new active sites and/or reduced long‐term stability.^[^ ^42^, ^43^, ^44^, ^45^ ^]^ However, such structural evolutions are often unpredictable and difficult to control, especially when compared to well‐defined, pre‐synthesized systems. These uncertainties in active site formation and stability present major challenges in the development of highly efficient and durable Ni‐based CO_2_ reduction catalysts. To better understand and mitigate these issues, further in‐depth studies on Ni–N–C catalysts are essential.

Herein, we synthesized Ni/NC catalysts for CO_2_RR using a simple milling‐pyrolysis method, wherein Ni particles were supported on nitrogen‐doped carbon. Upon electrochemical activation under an Ar atmosphere (Ar–Ni/NC), at – 0.8 V vs. RHE, the current density rose from ∼30 to ∼60 mA cm^−2^ (nearly doubling) while keeping CO selectivity at a high level of ∼90%. This enhancement suggests more active sites being present due to the transformation of Ni particles into atomically dispersed Ni single atoms during the activation process, thereby improving CO_2_RR performance. Control experiments further confirmed the essential role of Ni particles in the creation of active single ‐ atom sites. For instance, acid‐treated Ni/NC, which contained fewer Ni particles, exhibited significantly lower current density compared to both untreated Ni/NC and Ar–Ni/NC. This suggests that the absence of sufficient Ni particles limits the evolution of single atoms, leading to inferior catalytic activity. These comparative results highlight the importance of Ni particles as precursors for generating Ni single‐atom sites during activation, which is essential for achieving enhanced CO_2_RR performance.

## 2 Results and Discussion

**Figure 1:**
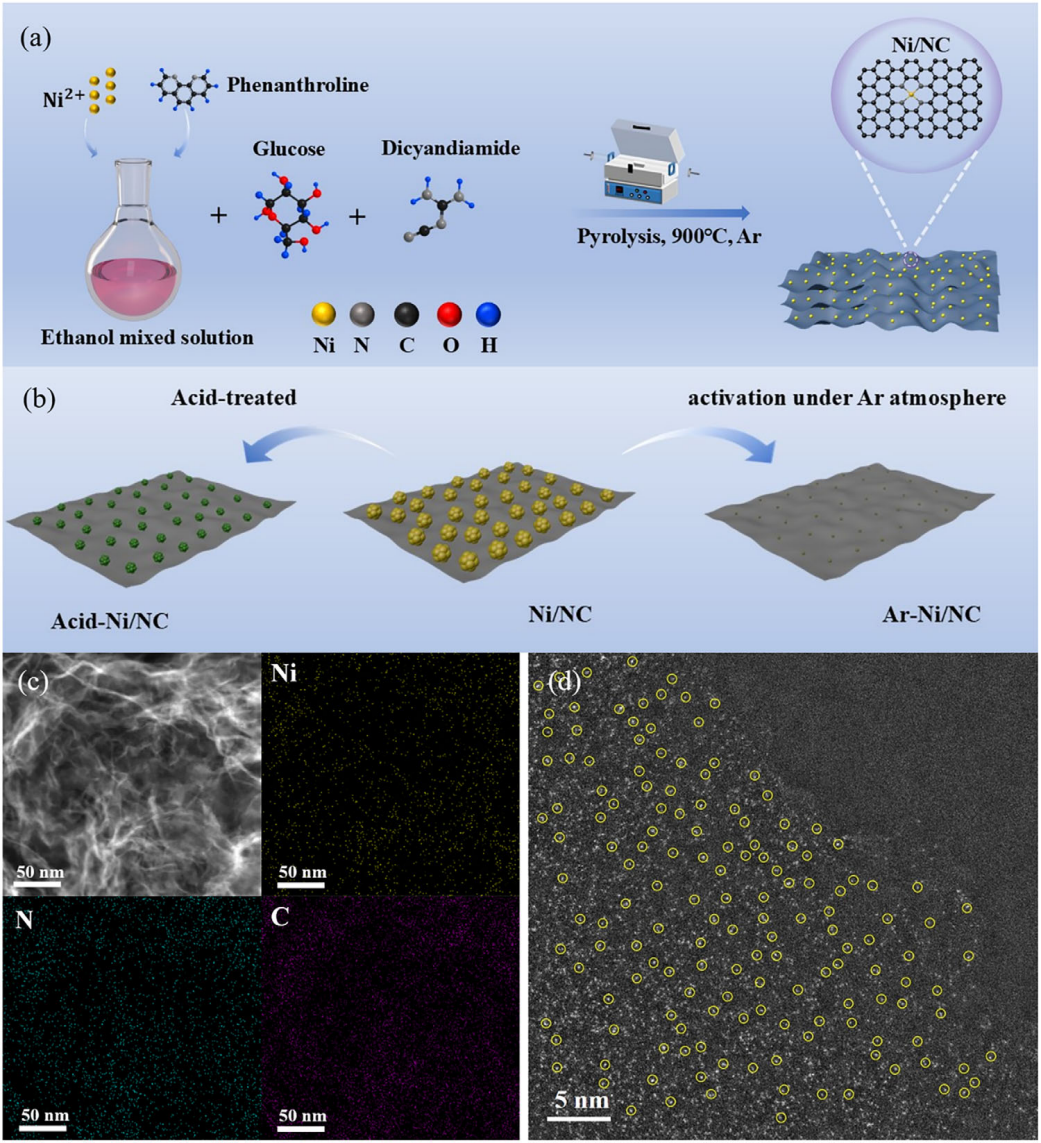
a) Schematic preparation of Ni/NC catalysts. b) Schematic diagram of the evolution of Ni single atoms from particles; c) HAADF‐STEM images (including corresponding EDS mappings of Ni, C, and N elements) of Ar‐Ni/NC; d) AC‐HAADF‐STEM image of Ar‐Ni/NC (the yellow circles are Ni single atoms).

The Ni/NC catalysts were prepared through grinding followed by pyrolysis, as described in **Figure** **1**a. Firstly, anhydrous nickel chloride and 1,10‐phenanthroline monohydrate were blended in ethanol to form a ligand structure. The resulting solution was then added to a pre‐milled mixture of glucose and dicyandiamide, followed by further milling to ensure homogeneity. Finally, the sample was subjected to annealing at 900 °C for 2 h under Ar, using a ramping rate of 2 °C min^−1^, to obtain the Ni/NC structure. In this structure, most of the Ni initially exists in the state of particles. However, after activation under Ar conditions, the Ni particles in Ni/NC transform into single‐atoms, while some Ni particles remain in Acid‐Ni/NC even after acid etching. The activation process and the evolution of single sites are illustrated schematically in Figure 1b. We performed scanning electron microscopy (SEM) and energy‐dispersive X‐ray spectroscopy (EDS) analyses to investigate the morphological and structural traits (Figures S1–S3, Supporting Information). SEM analysis reveals that Ni/NC features distinct, irregularly shaped Ni particles uniformly dispersed on the nitrogen‐doped carbon support. Following acid etching and activation under Ar atmosphere, a significant reduction in Ni particle content is observed. Notably, Ar activation results in a higher Ni loading compared to acid etching, as shown in Table S2 (Supporting Information). This suggests that Ar activation did not lead to a significant loss of Ni from the NC substrate, but rather resulted in a reduction in particle size, which may influence the CO_2_RR performance. We also performed transmission electron microscopy (TEM) to investigate the morphological features (Figure S4, Supporting Information). As seen in the TEM images of Ni/NC, well‐dispersed Ni nanoparticles are embedded within the N‐doped carbon layers. In contrast, the Ni nanoparticles in Acid‐Ni/NC appear partially etched, indicating structural degradation during acid treatment. The typical morphology of Ar‐Ni/NC features thin, layered structures without observable particles or clusters, indicating the transformation of nickel from nanoparticulate to single‐atom form.

Furthermore, the High ‐ Resolution Transmission Electron Microscopy (HR‐TEM) image of Ni/NC shows nickel particles with distinct, regularly arranged lattice fringes (Figure S5, Supporting Information). The specific lattice spacing of the (111) facet of the nickel particles in Ni/NC is measured to be 0.2115 nm. After acid etching, the lattice fringes turned blurred and were measured to be 0.2045 nm. Figure S7 (Supporting Information) displays the High‐Angle Annular Dark‐ Field Scanning Transmission Electron Microscopy (HAADF‐STEM) images and corresponding EDS elemental maps of Acid‐Ni/NC and Ni/NC, respectively. It is evident that in both catalysts, Ni particles exhibit good dispersion on the nitrogen‐doped carbon support. The most obvious difference is that the Ni content in Acid‐Ni/NC is significantly reduced. Additionally, the average particle sizes of Ni in Ni/NC and acid‐etched Ni/NC were determined to be 6.92 and 1.43 nm, respectively (Figure S6, Supporting Information), indicating a substantial decrease in particle size following acid treatment. Upon activation under Ar conditions, the distribution of Ni was retained, as evidenced by the HAADF‐STEM images and corresponding EDS mappings shown in Figure 1c and Figure S8 (Supporting Information). Afterwards, aberration‐corrected high‐angle annular dark‐field scanning transmission electron microscopy (AC‐HAADF‐STEM) provides additional confirmation of nickel species existing in the Ar‐Ni/NC catalyst. As shown in Figure 1d and Figure S9 (Supporting Information), no nickel particles in the tens‐of‐nanometers range are observed. Instead, isolated bright spots uniformly distributed across the carbon matrix are visible, indicating the presence of individual nickel atoms and confirming the transformation of the particles into a single‐atom configuration. Moreover, the enlarged AC‐HAADF‐STEM images and their corresponding EDS maps (Figure S10, Supporting Information) show that Ni, N, and O are homogeneously distributed on the carbon support.

**Figure 2:**
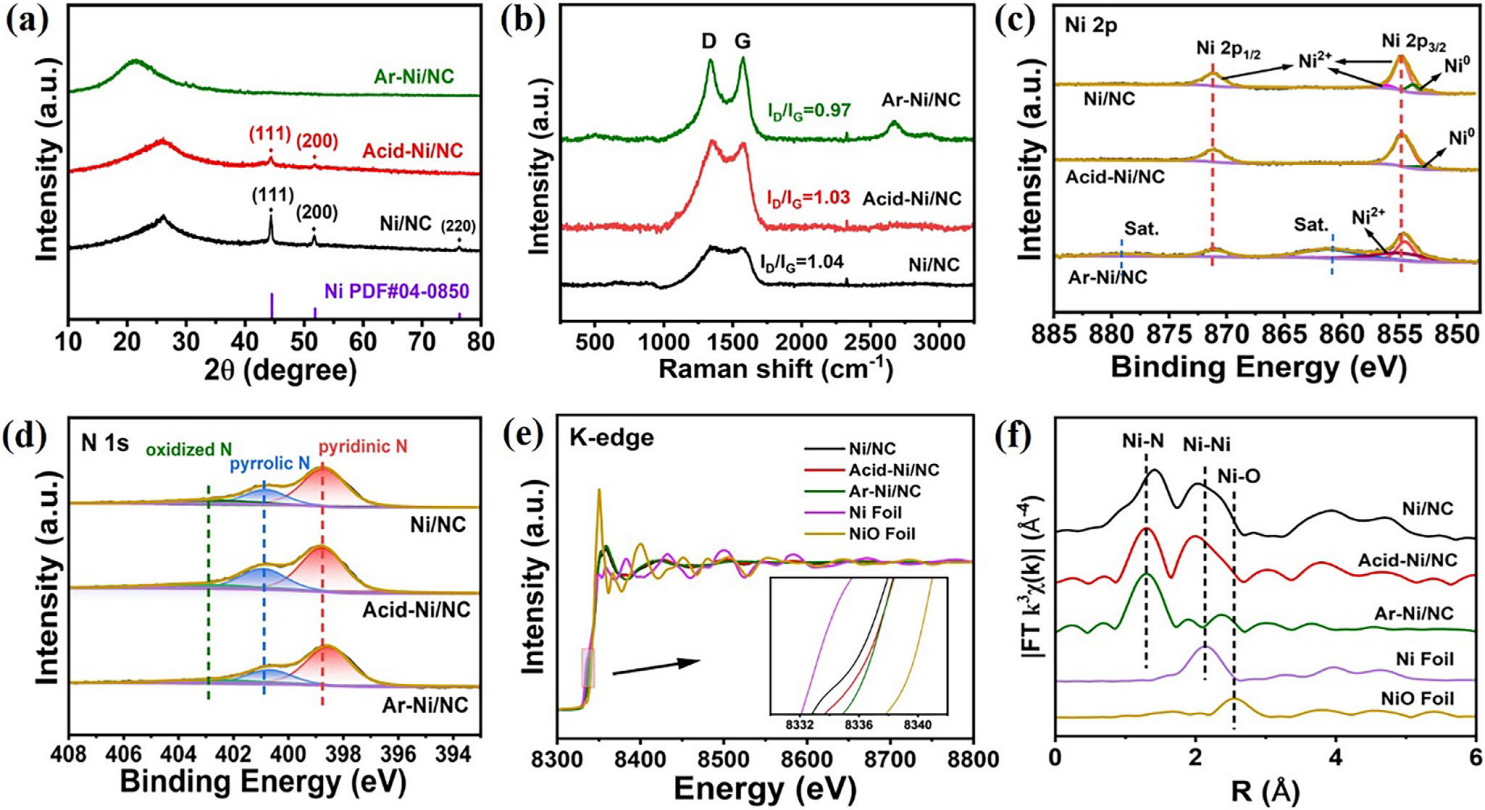
a) XRD patterns, b) Raman spectra, c) Ni 2p spectra, d) N 1s high‐resolution spectra of various Ni/NC catalysts; e) Ni K edge XANES spectra of Ni/NC, Acid‐Ni/NC, Ar‐Ni/NC, Ni Foil and NiO Foil (the inset is the magnified image); f) Fourier transformation (FT) of the EXAFS spectra.

The XRD pattern of Ni/NC exhibits three clear diffraction peaks at 44.3°, 51.5°, and 76.3°, with each peak corresponding to the Ni (111), Ni (200), and Ni (220) facets in sequence (**Figure** **2**a). After acid treatment, the XRD pattern of Acid‐Ni/NC retains the Ni (200) and Ni (220) peaks, but with reduced intensity, suggesting partial removal or dispersion of Ni species. However, no significant Ni‐related peaks are observed in Ar‐Ni/NC, indicating the transformation of Ni into single atoms after activation. Additionally, no peaks corresponding to nitrides were detected, confirming the successful achievement of N atom doping into the carbon skeleton. The Raman spectra (Figure 2b) show two typical signals at 1352 cm^−1^ (D band) and 1586 cm^−1^ (G band).^[^ ^42^, ^46^ ^]^ The ratios of D peak area to G peak area (I_D_/I_G_) for all the prepared catalysts are close to one, indicating that they have a similar level of graphitization and defects. These results suggest that an increase in graphitization would be beneficial in improving the electrical conductivity and catalytic activity of the materials. We used X ‐ ray photoelectron spectroscopy (XPS) to investigate the chemical valence states and surface compositions of the prepared catalysts. In the Ni 2p spectra (Figure 2c), two prominent peaks corresponding to Ni 2p_3/2_ (855.1 ± 0.1 eV) and Ni 2p_1/2_ (872.3 ± 0.2 eV) are observed. Additionally, the presence of a peak at 852.7 eV, characteristic of metallic Ni⁰, was observed in both Ni/NC and Acid‐Ni/NC catalysts, indicating that nickel exists in the form of nanoparticles.^[^ ^47^, ^48^, ^49^, ^50^, ^51^ ^]^ However, after activation under an Ar atmosphere, the Ar‐Ni/NC catalyst only exhibits two satellite peaks at binding energies of around ≈861.7 and 880.5 eV, matching the Ni^2^⁺ state, indicating a high dispersion of Ni species. The N 1s XPS spectra can be deconvoluted into three characteristic peaks, which are pyridinic N (398.6 ± 0.1 eV), pyrrolic N (400.7 ± 0.1 eV), and oxidized N (403 ± 0.1 eV) in sequence.^[^ ^52^, ^53^, ^54^ ^]^ This shows that the nitrogen environment remains stable in all catalysts, meaning acid treatment and activation do not change the nitrogen sites much (Figure 2d; Table S1, Supporting Information). The C 1s spectra demonstrate the successful doping of N atoms into the carbon skeleton (Figure S11, Supporting Information).^[^ ^55^, ^56^ ^]^ O 1s spectra show an upward shift in oxygen vacancy binding energy for Ar‐Ni/NC, signifying electron‐deficient oxygen species with higher valence states, thereby boosting catalytic activity and structural integrity (Figure S12, Supporting Information). In addition, through Fourier transform infrared (FT ‐ IR) spectroscopy, a broad absorption band is detected in the 3000 to 3680 cm^−1^ range, attributed to N─H bonds and the stretching vibrations of surface‐adsorbed water molecules. Broad peaks observed between 1100 and 1700 cm^−1^ correspond to C─N bonds, suggesting the incorporation of nitrogen into the carbon framework and indicating potential synergistic effects within the carbon network (Figure S13, Supporting Information).^[^ ^57^, ^58^, ^59^ ^]^ To obtain a more in‐depth understanding of the bonding properties and coordination environment of Ni in Ni/NC, Acid ‐ Ni/NC, and Ar ‐ Ni/NC, X ‐ ray absorption spectroscopy (XAS) was performed. The Ni K ‐ edge X ‐ ray absorption near ‐ edge structure (XANES) spectra of the prepared catalysts, along with reference Ni foil and NiO foil, are presented in Figure 2e. As shown in Figure 2e, the absorption edge energies of Ni/NC, Acid‐Ni/NC, and Ar‐Ni/NC catalysts shift toward higher energies compared to Ni foil, indicating that Ni exists in a higher oxidation state. In addition, compared to NiO (a typical oxide environment with Ni^2^⁺), Ar – Ni/NC shows a slightly elevated absorption edge, implying that the Ni^2^⁺ state in Ni–N_x_ sites is more electron‐deficient, which is consistent with their stronger ability to activate CO_2_ to ^*^COOH.^[^ ^60^ ^]^ For a more in ‐ depth determination of the catalyst’s coordination structure, Ni K ‐ edge X ‐ ray absorption fine structure (EXAFS) analysis was performed as well. Figure 2fshows that the peak at roughly 2.15 Å for the Ni/NC and Acid ‐ Ni/NC catalysts has a near ‐ edge structure comparable to that of Ni foil, implying the existence of Ni nanoparticles; this is in agreement with the XRD results. In contrast, no peak associated with Ni–Ni interactions, typically observed at ≈2.15 Å in Ni foil, was observed in the Ar‐Ni/NC spectrum, indicating the absence of Ni nanoparticles. Meanwhile, no Ni‐O scattering peak (≈1.3 Å) was detected, ruling out the contribution of oxides to the catalytic activity. Instead, a prominent signal peak at ≈1.3 Å, corresponding to the Ni–N scattering path, was observed in the Ar‐Ni/NC catalysts. These results confirm that isolated Ni–N_x_ sites are evenly distributed on the hierarchical porous carbon support.^[^ ^61^ ^]^

**Figure 3:**
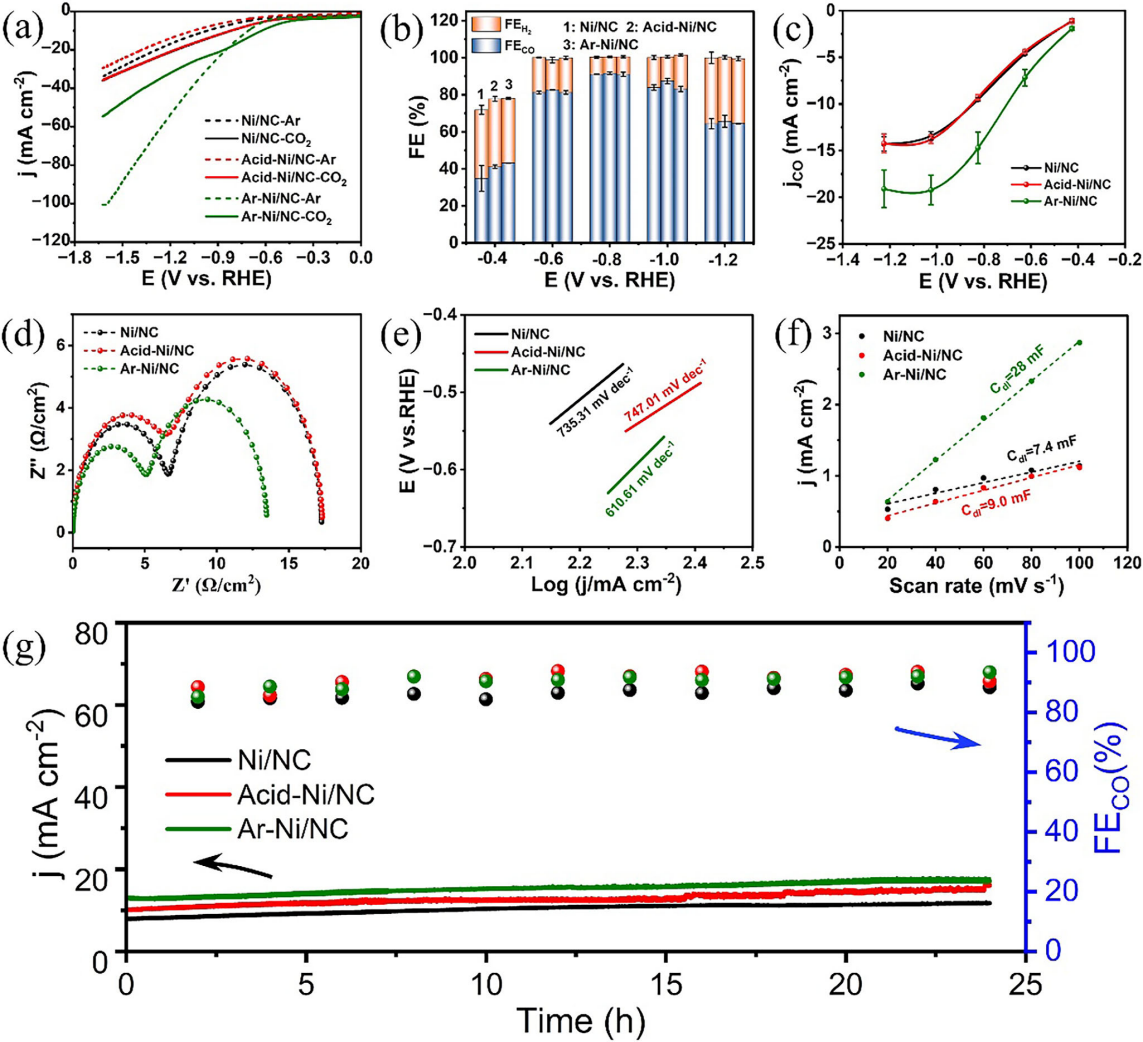
a) LSV curves; b) FE_CO_; c) CO partial current densities; d) Nyquist plots; e) Tafel plots and f) Electrochemical double‐layer capacitance of various Ni/NC catalysts in CO_2_‐saturated 0.1 M KHCO_3_; g) Durability test of Ni/NC, Acid‐Ni/NC and Ar‐Ni/NC catalysts at −0.8 V (vs. RHE) for 24 h.

Electrochemical tests were performed in an H‐type cell using a CO_2_ ‐ saturated 0.1 M potassium bicarbonate (KHCO_3_) electrolyte to evaluate the CO_2_RR performance of the as‐synthesized catalysts (Figure S18, Supporting Information).^[^ ^62^ ^]^ The catalytic activity and selectivity of Ni/NC, Acid‐Ni/NC, and Ar‐Ni/NC were systematically assessed via chronoamperometric (CA) measurements, while CO production was quantified using gas chromatography (GC). Prior to CO_2_RR testing, the catalysts were activated under Ar‐saturated conditions to generate the desired active sites (Figure S14, Supporting Information). During this activation step, higher current densities were observed under Ar‐saturated conditions, likely due to the enhanced number of Ni active sites. **Figure** **3**a displays the linear sweep voltammetry (LSV) curves of all catalysts under both Ar‐saturated and CO_2_‐saturated conditions. Notably, Ar‐Ni/NC displayed greater current densities than Ni/NC and Acid‐Ni/NC, suggesting the in situ formation of more atomically dispersed Ni single sites that enhance CO_2_RR activity. Following the LSV measurements, the CO_2_RR selectivity of each catalyst was evaluated at various applied potentials. As shown in Figure 3b, all catalysts displayed high selectivity toward CO formation with significantly suppressed H_2_ evolution across a broad potential range. For instance, at −0.8 V (vs. RHE), the Faradaic efficiency for CO (FE_CO_) exceeded 80% for all catalysts. Although all the catalysts exhibited good selectivity, Ar‐Ni/NC achieved a higher current density at a lower potential. Moreover, it delivered the highest CO partial current density (j_CO_), demonstrating superior catalytic performance (Figure 3c). These results confirm that the optimized Ar‐Ni/NC catalyst demonstrates outstanding CO_2_RR activity and selectivity, a performance that can be ascribed to the successful generation of more atomically dispersed Ni active sites.

To delve deeper into the CO_2_RR kinetics, electrochemical impedance spectroscopy (EIS) measurements were conducted. As shown in the Nyquist plots (Figure 3d), the interfacial charge‐transfer resistance (R_ct_) of Ar‐Ni/NC was notably lower than that of the other two catalysts, indicating its more efficient and faster charge‐transfer process during CO_2_RR.^[^ ^63^ ^]^ The accelerated charge transfer observed in Ar‐Ni/NC can be attributed to the formation of more atomically dispersed Ni single sites during the activation process, and consequently, leads to an enhanced CO_2_RR performance. In addition, Tafel slope analysis was performed to assess the reaction kinetics of CO_2_RR over these three catalysts. As shown in Figure 3e, the Tafel slope of Ar‐Ni/NC (610.61 mV dec^−1^) is notably lower than those of Ni/NC (735.31 mV dec^−1^) and Acid‐Ni/NC (747.01 mV dec^−1^). The decreased Tafel slope suggests that the electron transfer process is more efficient and the energy barrier for intermediate formation is lower. Based on the above, it can be concluded that the key role of electrochemical activation is to redisperse Ni nanoparticles into atomically dispersed Ni single sites, thereby accelerating the CO_2_RR.^[^ ^64^, ^65^ ^]^

To explore the origin of the Ar ‐ Ni/NC catalyst’s excellent catalytic activity in more detail, the double ‐ layer capacitance (C_dl_) was used to estimate its electrochemical active surface area (ECSA), which was subsequently compared with that of Ni/NC and Acid ‐ Ni/NC (Figure 3f; Figures S15–S17, Supporting Information). The Ar‐Ni/NC catalyst exhibits a higher double‐layer capacitance (C_dl_), while the current density of j_ECSA_ is very low, which indicates that the CO_2_RR process is governed not by the surface area (the quantity of active sites) but by the activity of each individual site in the catalyst.^[^ ^66^ ^]^ On the other hand, the catalytic performance of Ar‐Ni/NC tested in the H‐cell was comparable to that of previously reported Ni single‐atom catalysts (SACs), as summarized in Table S3 (Supporting Information), demonstrating its competitive CO_2_RR activity. Furthermore, the stability of Ni/NC, Acid‐Ni/NC, and Ar‐Ni/NC catalysts was evaluated through continuous electrolysis at −0.8 V (vs. RHE), as shown in Figure 3g. For 24 hours, the current density and FE_CO_ remained almost constant, and Ar ‐ Ni/NC exhibited a significantly higher current density compared to Ni/NC and Acid ‐ Ni/NC, which verifies the outstanding long ‐ term stability of the Ar ‐ Ni/NC catalyst during CO_2_RR.

**Figure 4:**
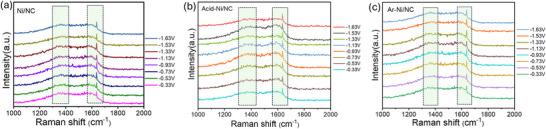
In situ Raman spectra collected at different processes under CO_2_RR for Ni/NC, Acid‐Ni/NC and Ar‐Ni/NC. All potentials (−0.33, −0.53, −0.73, −0.93, −1.13, −1.33, −1.53, and −1.63 V) were calibrated to the RHE scale. In situ Raman spectra of a) untreated Ni/NC, b) acid‐treated Ni/NC, and c) Ar‐Ni/NC catalysts.

To gain insights into the catalytic mechanism and identify surface‐adsorbed intermediates during CO_2_ reduction, we conducted in situ Raman spectroscopy at various applied potentials (**Figure** **4**; experimental setup in Figure S19, Supporting Information).^[^ ^67^, ^68^, ^69^ ^]^ As shown in Figure 4, all three catalysts exhibit vibrational bands at ≈1380 and ≈1637 cm^−1^, corresponding to the C─O and C═O stretching modes of the ^*^COOH intermediate, respectively, confirming this as the key reaction pathway.^[^ ^70^ ^]^ Notably, Ni/NC and Acid‐Ni/NC showed unchanged peak intensities at more negative potentials, indicating stable ^*^COOH coverage. In contrast, Ar‐Ni/NC exhibited a slight increase in intensity, suggesting enhanced ^*^COOH adsorption/stabilization, which aligns with its superior electrochemical performance. No ^*^CO features were detected, reinforcing ^*^COOH as the dominant intermediate. XANES (Figure 2e) and EXAFS (Figure 2f) analyses revealed Ar‐Ni/NC contains Ni^2^⁺ in Ni‐N_x_ sites (no Ni‐Ni interactions) with higher oxidation states than Ni particles. This electron‐deficient Ni^2^⁺ enhances electron donation to CO_2_, facilitating ^*^COOH formation (CO_2_ + e^−^ + H⁺ → ^*^COOH). Strong Ni‐N coordination modulates Ni's d‐orbital density, optimizing overlap with CO_2_ antibonding orbitals and lowering activation barriers. AC‐STEM‐confirmed absence of Ni nanoparticles reduces steric hindrance, while NC's pyridinic/pyrrolic N (Figure 2d) stabilizes ^*^COOH via hydrogen/electrostatic interactions.^[^ ^71^, ^72^, ^73^, ^74^, ^75^ ^]^

## 3 Conclusion

In this study, we present a simple approach to modulate the number of atomically dispersed Ni single sites in Ni‐N‐C‐based materials through Ar activation, resulting in an increase in current density from ≈30 to 60 mA cm^−^^2^ while maintaining a high selectivity (≈90%) for CO production. The activation process plays a pivotal role in redistributing Ni particles into atomically dispersed Ni single sites, as confirmed by XPS, EXAFS, and AC‐STEM analyses. Furthermore, the presence of nitrogen atoms in different environments is crucial for stabilizing the redispersed Ni single atoms, facilitating the formation of Ni─N bonds. Through in situ Raman spectroscopy, it is observed that the conversion of CO_2_ to CO involves the generation of the *COOH intermediate. This study highlights the gradual structural reconstruction of Ni particle catalysts supported on N‐C substrates during electrochemical CO_2_ reduction, contributing to improved catalytic performance. These findings emphasize the significance of structural evolution in catalysts and offer a promising route for designing more efficient M‐N‐C catalysts.

## Conflict of Interest

The authors declare no conflict of interest.

## Supporting information



Supporting Information

## Data Availability

The data that support the findings of this study are available from the corresponding author upon reasonable request.
