# Supplementary material for: Directed Structural Evolution of Nickel Nanoparticles into Atomically Dispersed Sites for Efficient CO2 Electroreduction
Source: Small. 2025 Sep 5;21(40):e05521. doi: 10.1002/smll.202505521 (PMC12508711; doi:10.1002/smll.202505521)
Supplement: Supplementary file 1 — Supporting Information [file SMLL-21-e05521-s001.docx]

**Supporting Information**

**Directed Structural Evolution of Nickel Nanoparticles into Atomically Dispersed Sites for Efficient CO₂ Electroreduction.**

Xiao Li,^1┴^ Tao Gan,^2┴^ Xinhua Gao, ^1┴^ Bing Li, ^1^ Juan Peng, ^1^ Yang Ji, ^1^ Shenghua Chen,^3^ Jian Zhang,^4^ Junjun Zhang, ^1^ Pradip Kumar Das,^5^ Vinoth Ramalingam,^6^ Maolin Zhang,^7^* Pengfei Zhang,^1,8^* Karthik Peramaiah,^5^* Yajun Qiu^1^*

^1^State Key Laboratory of High-efficiency Utilization of Coal and Green Chemical Engineering, College of Chemistry and Chemical Engineering, Ningxia University, Yinchuan, Ningxia 750021, P. R. China

^2^Shanghai Synchrotron Radiation Facility, Shanghai Advanced Research Institute, Chinese Academy of Sciences, Shanghai 201204, P. R. China

^3^School of Chemistry, National Innovation Platform (Center) for Industry-Education Integration of Energy Storage Technology, Xi'an Jiaotong University, Xi'an, 710049, P. R. China

^4^Key Laboratory of Carbon Materials of Zhejiang Province, Key Lab of Biohealth Materials and Chemistry of Wenzhou, College of Chemistry and Materials Engineering, Wenzhou University, Wenzhou, Zhejiang 325035, P. R. China

^5^Institute of Institute of Sustainability for Chemicals, Energy, and Environment (ISCE2), Agency for Science, Technology and Research (A*STAR), 1 Pesek Road, Singapore, 627833, Singapore

^6^School of Computing, Engineering and Technology, Robert Gordon University, Garthdee Road, Aberdeen, AB10 7GJ, UK

^7^Institute of Environment and Sustainable Development in Agriculture, Chinese Academy of Agricultural Sciences, Beijing, 100081, P. R. China

^8^School of Chemistry and Chemical Engineering, Shanghai Jiao Tong University,

Shanghai, 200240, P. R. China

^┴^These authors contributed equally: Xiao Li, Tao Gan and Xinhua Gao.

**1. Experimental section**

1.1 Chemicals

All the chemicals were used without further purification.

Nickel(Ⅱ)Chloride, Anhydrous(Cl_2_Ni, Ni＞42%, Damas-beta), 1,10-Phenanthroline Monohydrate(C_12_H_8_N_2_·H_2_O, AR, 98%, Aladdin), D-(+)-Glucose(≥99.5%, Aladdin), Dicyandiamide(AR, Macklin), potassium bicarbonate (KHCO_3_≥99.5%), Hydrochloric Acid(HCl, 36%~38%), potassium hydroxide(KOH, AR≥99.5%) Ethanol absolute(AR≥99.5%), Nafion solution (5 wt%) were purchased from Sigma-Aldrich. Deionized (DI) water used in our experiments was supplied by LabSE-Q Deionisation Water Purifier (Shanghai, ZHIANG).

**1.2 Material Characterization**

The X-ray diffraction (XRD) patterns were recorded on a Rigaku MiniFlex 600-C X-ray diffractometer with Cu-Kα radiation with a scan rate of 10° min^-1^. The Raman measurement was performed with Thermo Fisher Spectrometer from American, wavenumber region 9-3600 cm^-1^. FTIR spectroscopy was carried out on PerkinElmer FT-IR Spectrometer Spectrum 3 with the spectral range of 4000-400 cm^-1^.

K-Alpha ray photoelectron spectroscopy (XPS) analysis was performed on AXIS SUPRA^+^ instrument. In situ Raman spectra were performed using the Labram Soleil Raman spectrometer (HORIBA Jobin Yvon) with the laser of λ = 532 nm. The In-situ Raman electrochemical reaction cell purchased from the CIS-Raman-EC-U2-H from the manufacturer of In-situ High-Tech. The data of XAFS spectra at the Ni K-edge were collected at the beamline BL14W1 station of the Shanghai Synchrotron Radiation Facility (SSRF), China. The morphology of the samples was determined by scanning electron microscopy (Axia ChemiSEM) and transmission electron microscopy (TEM, HT7700, 200 kV).

**1.3 Material synthesis**

**1.3.1 Synthesis of Ni/NC**

The specific synthesis process of Ni/NC is described as follows. First, 129.6 mg of Nickel(Ⅱ)Chloride, Anhydrous was dissolved in 3 ml of ethanol solution, and then 1.586 g of 1,10-Phenanthroline Monohydrate was added to the above solution and dispersed homogeneously to form a mixed solution. Next, 300 mg of D-(+)-Glucose and 12 g of Dicyandiamide were mixed and ground in a mortar until powdered, and then the mixed solution was added into this mortar and continued to be ground to make a homogeneous mixture of the drugs. Finally, the sample was annealed at 900 °C for 2h under an argon atmosphere with a ramping rate of 2 °C min^-1^ to obtain the final product.

**1.3.2 Synthesis of Acid-Ni/NC**

Apply etchant (4 M HCl aqueous solution) and maintain at 60 ℃ for 12 h. The procedure was as follows: Firstly, 150 mg of Ni-900 was put into a 50 ml centrifuge tube, 4 M HCl aqueous solution was added and dispersed homogeneously by ultrasonication. It was then sealed and placed on an agitating heater and kept at 60°C with heating and stirring for 12h. The as-obtained precipitates were centrifuged and washed with deionized water three times and once with ethanol and dried at 60 ℃ overnight.

**1.3.3 Synthesis of Ar-Ni/NC**

This catalyst is mainly based on the Ni/NC catalyst activated by electrochemical CV with an Ar-saturated atmosphere for 10h. The synthesis procedure is as follows: First, the electrodes were prepared by dropping Ni/NC onto carbon paper, and then subsequent operations were performed in an H-cell. Ar-saturated atmosphere was introduced with the flow rate set at 20 ml min^-1^, activated by CV in the range of -0.5 to -2.4 V for 10h, and then LSV was measured in this range, and then CO_2_-saturated atmosphere was introduced with the gas flow rate set at 20 ml min^-1^, and then LSV was measured after 15 revolutions of activation by CV in the above voltage range, and then then electrolyzed at each of these five potentials (-1.2 V, -1.4 V, -1.6 V, -1.8 V, and -2.0 V vs. SCE) for 1 h 20 min. Finally, the sample on the carbon paper was dried with an infrared lamp, and its sample was gently scraped off and collected; this sample was named as Ar-Ni/NC.

**1.4 Electrochemical measurements**

Preparation of working electrode: For the H-type cell, the electrode preparation was as follows: 5 mg of the sample was dispersed in the mixed solution of 720 μL of ethanol (0.72 ml), 250 μL of DI water, and 30 μL of Nafion solution (5 wt%), followed by sonication for at least 30 min to obtain homogeneous ink. Subsequently, a measure of 100 μL of the ink was drop-cast onto carbon paper (loading density=0.5 mg cm^-2^), and dried under an infrared light.

In this study, all potentials were calibrated against the hydrogen electrode. By the following formula: E_RHE_ = E_SCE_ + 0.2415 + 0.0592 × pH.

Electrochemical testing in an H-cell using a bipolar membrane on a CHI760E electrochemistry workstation in a three-electrode system. Using 1 cm^2^ carbon paper as the loading area for the preparation of working electrodes. The saturated calomel electrode (SCE) and platinum sheet were used as the reference and counter electrodes, CO_2_-saturated 0.1 M KHCO_3_ as catholyte, and 1 M KOH solution was used as anolyte. The samples are then electrochemically tested. Before testing, saturation with high-purity CO_2_ gas was performed for at least 20 min, and the flow rate was calibrated to 20 sccm using a mass flow controller and stirred with a magnetic stirrer to accelerate mass transfer. Then, CV curves and LSV curves were sequentially acquired over the voltage range of 0.5 V to -2.4 V vs. SCE at a scan rate of 50 s^-1^. In addition, an electrochemical impedance spectroscopy (EIS) test (Figure 3d) was performed at -0.6 V (vs. RHE) with the frequency ranging from 1 Hz to 100 kHz. The electrochemical active surface area (ECSA) was plotted by testing the CV at different scan rates (20, 40, 60, 80 and 100 mV s^-1^) and then deriving the ECSA from the electrochemical capacitance (C_dl_) (Figure 3f) and thus calculating the $j_{\mathrm{ECSA}}$ (Figure S16) for the LSV test voltage interval. The high ECSA of Ni/NC, Acid-Ni/NC, and Ar-Ni/NC could be attributed to the Ni-N_x_ structure, which exposes numerous active sites and further enhances the CO_2_RR performance. Constant potential electrolysis was carried out at different potentials for 1 h 20 min to analyze the products. Meanwhile, the Faradaic efficiency (FE) of gaseous products was calculated using the following equation:

$$\mathrm{FE}_{g}=\frac{n_{e}\times x\times F\times flowrate\times P}{R\times T\times I}$$

Where $n_{e}$ Is the number of electron moles required to obtain 1 molecule of product, $x$ is the ppm of the gaseous product formed, $F$ is the Faraday constant for 96485 C mol^-1^ , $\mathrm{flowrate}$ is the flow rate controlled by a mass flow controller (20 sccm), $P$ is the atmospheric pressure (101325 Pa), $R$ is 8.314 J K^-1^ mol^-1^, $T$ is 298.15 K, $I$ is the average current during the time of sampling.

**1.5 In situ Raman measurement**

In situ Raman spectra were performed using the Labram Soleil Raman spectrometer (HORIBA Jobin Yvon) with the laser of λ = 532 nm. The voltage and current signals are collected in a customized configuration controlled by an electrochemical workstation (CHI760). The three-electrode system was also used, where a Pt wire ring and an SCE electrode were used as the counter electrode and reference electrode, respectively. The catalyst was dropped on the carbon paper electrode as the working electrode. 1 M KOH solution was used as anolyte. The cathode electrolyte was the CO_2_-saturated 0.1 M KHCO_3_ solution, prepared by bubbling CO_2_ for at least 30 min. The electrolyte flow rate was kept at 20 mL min^−1^. The CO_2_RR tests were conducted for the four Ni catalysts in this study at different potentials from OCP to −1.6 V vs. RHE. The Raman results were collected in the wavelength range of 50 to 4000 cm^-1^.


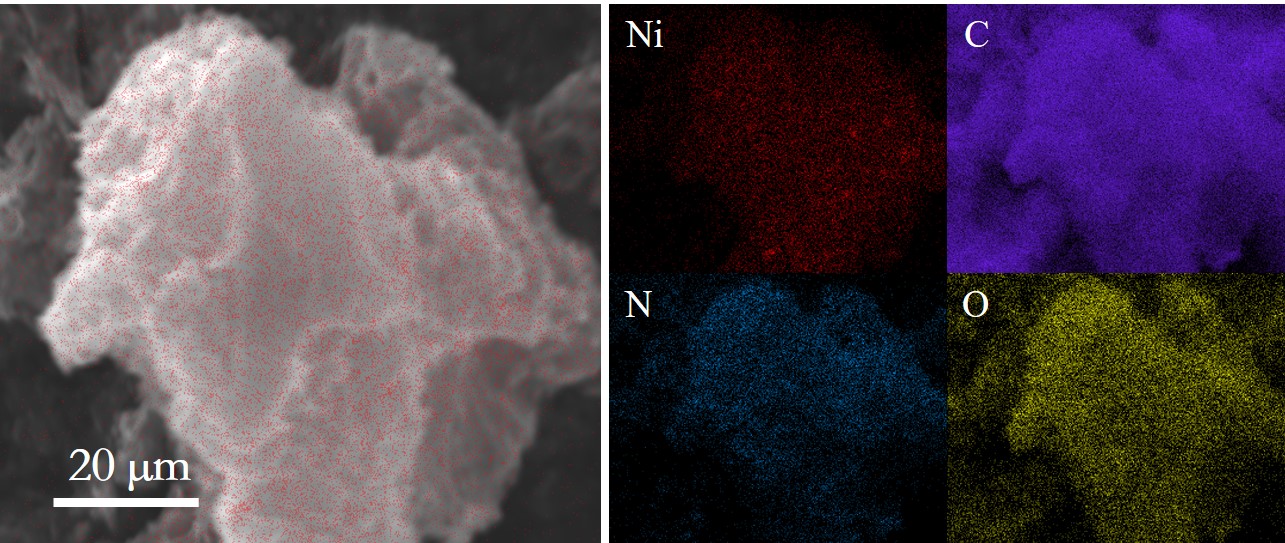


**Figure S1.** SEM images of Ni/NC and the corresponding element mapping images of Ni, N, C and O.

SEM shows that Ni/NC is a lamellar structure and shows Ni particles in Ni/NC uniformly loaded on nitrogen doped carriers, and EDS mappings confirmed that Ni, N, C, and O elements were homogeneously distributed.


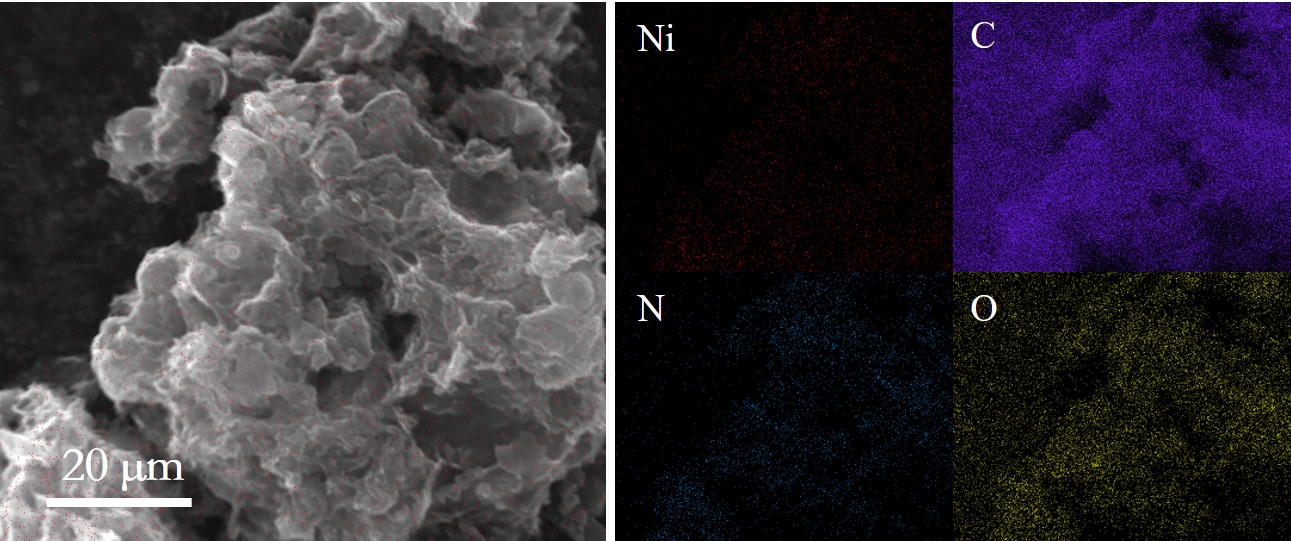


**Figure S2.** SEM images of Acid-Ni/NC and the corresponding element mapping images of Ni, N, C and O.

SEM shows that Acid-Ni/NC is a lamellar structure and shows fewer Ni particles in Acid-Ni/NC compared to Ni/NC and EDS mappings confirmed that Ni, N, C, and O elements were homogeneously distributed.


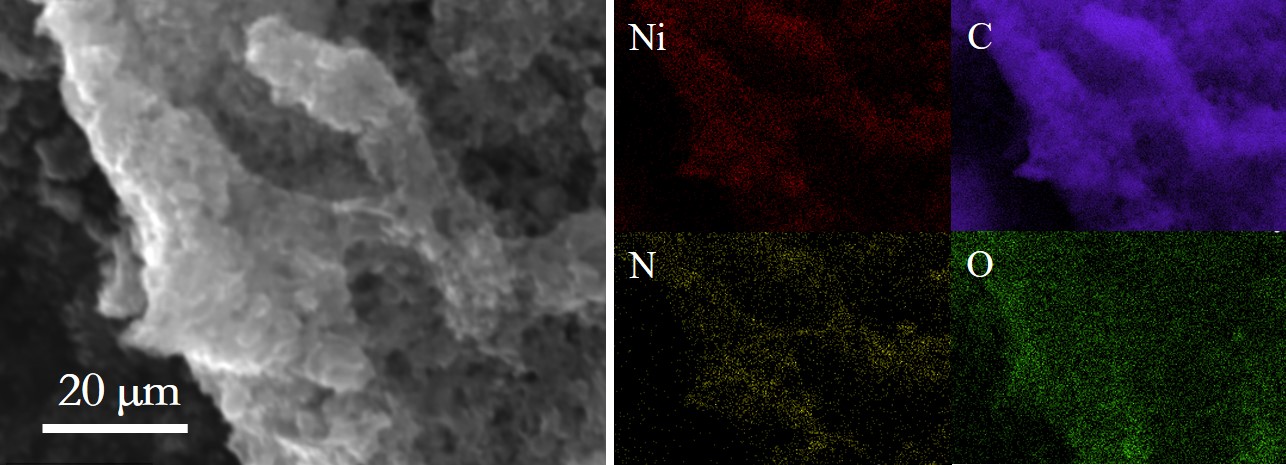


**Figure S3.** SEM images of Ar-Ni/NC and the corresponding element mapping images of Ni, N, C and O.

SEM and corresponding EDS images of Ar-Ni/NC show that nickel is uniformly distributed on the nitrogen doped carbon carriers.


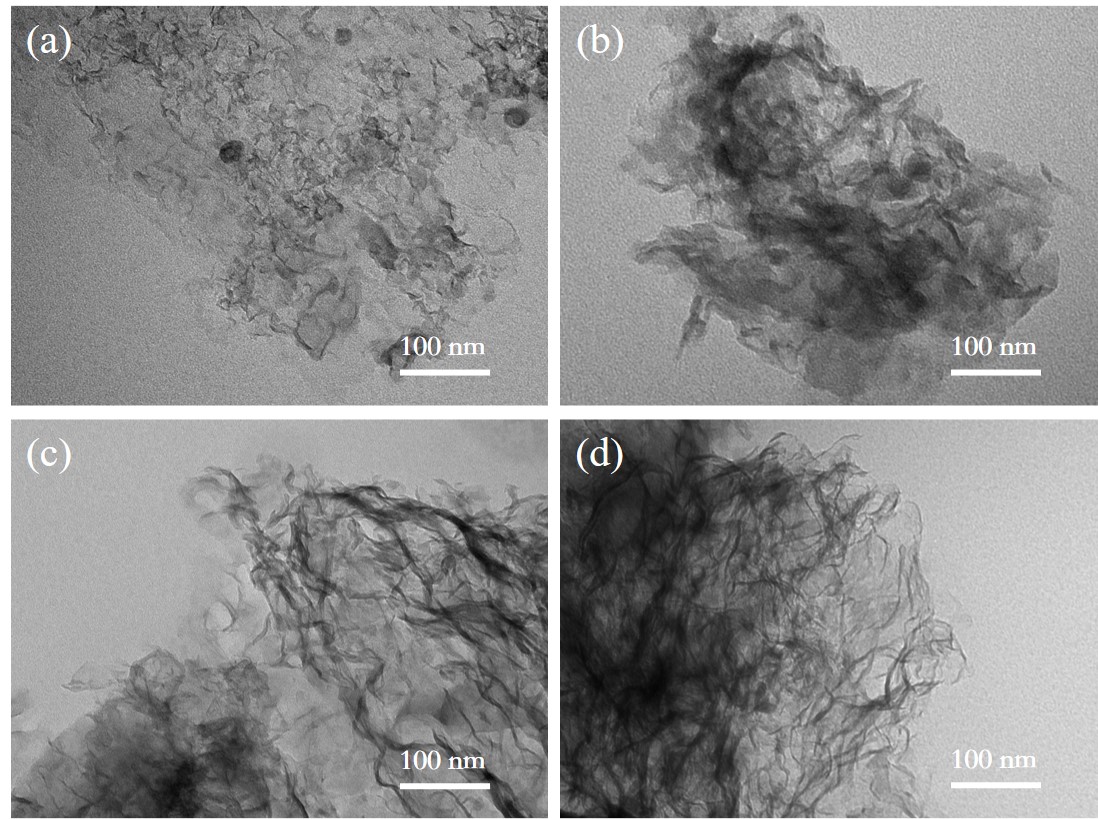


**Figure S4.** TEM images of (a) Ni/NC, (b) Acid-Ni/NC, (c) and (d) Ar-Ni/NC.

TEM images show that nickel in Ni/NC and Acid-Ni/NC exists in particulate form, and nickel in Ar-Ni/NC may exist in monoatomic form.


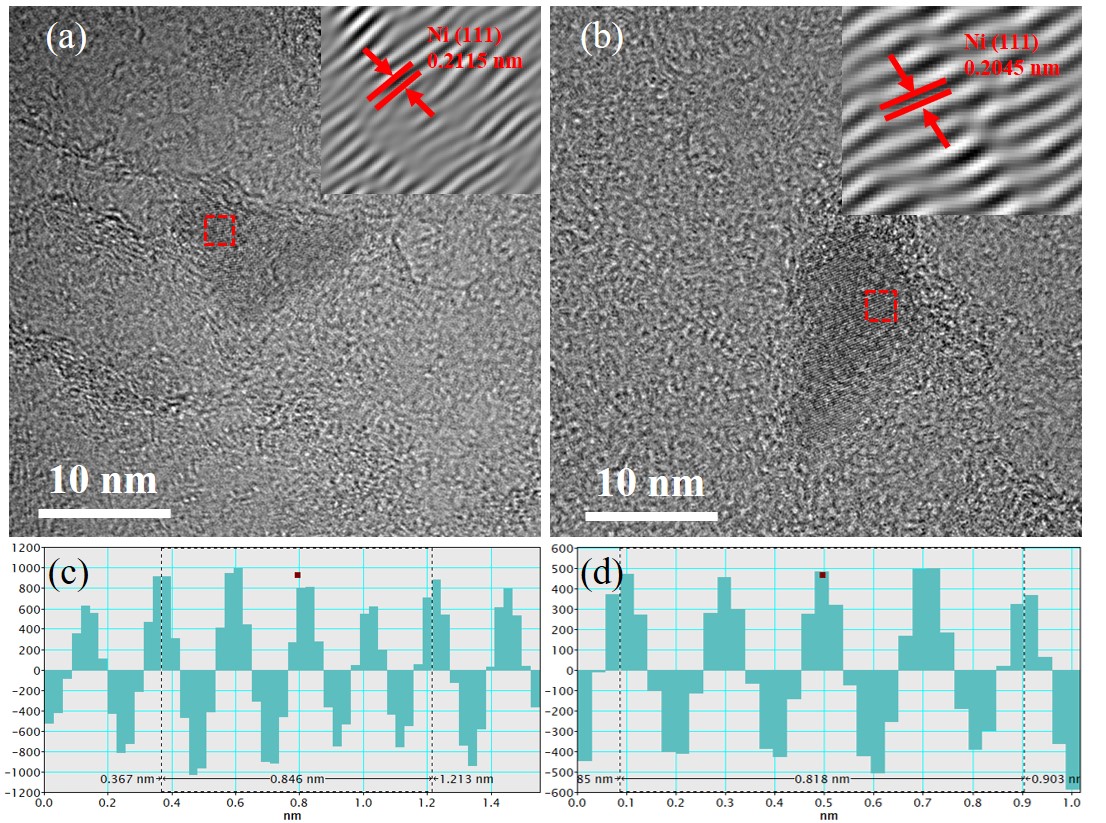


**Figure S5.** (a) The lattice spacing d = 0.2115 nm under the Ni (111) crystal plane of Ni/NC under High-resolution TEM (HRTEM) images. (b) The lattice spacing d = 0.2045 nm under the Ni (111) crystal plane of Acid-Ni/NC under High-resolution TEM (HRTEM) images. The Fourier transform plots of the lattice spacing of Ni elements under high-resolution electron microscopy for (c) Ni/NC and (d) Acid-Ni/NC, respectively.


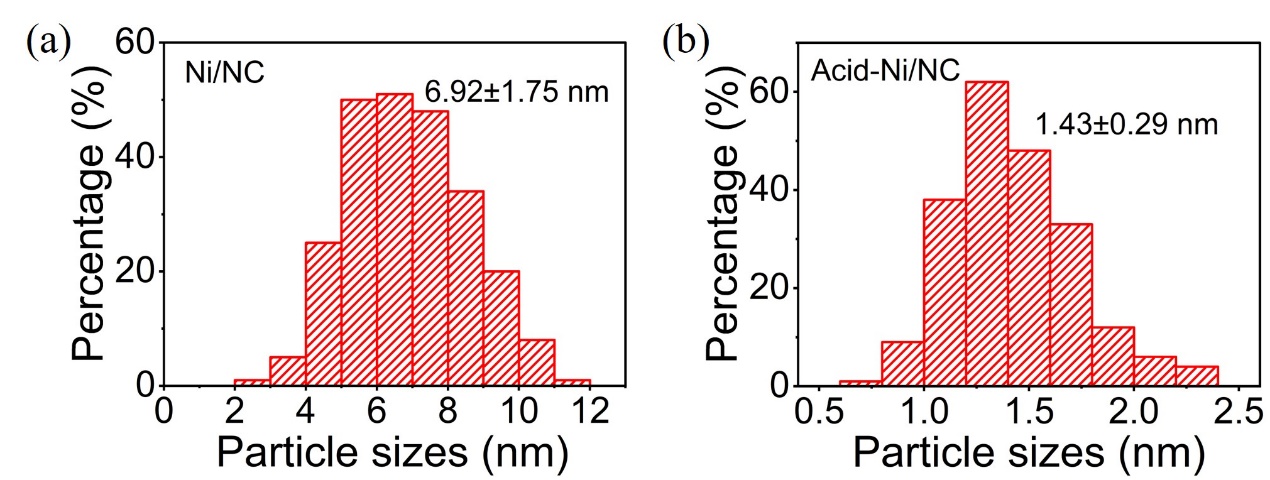


**Figure S6.** Ni particle size distribution of (a) Ni/NC and (b) Acid-Ni/NC.

From the above particle size diagram, it can be seen that the Ni particle size in Ni/NC is significantly larger than that in Acid-Ni/NC.


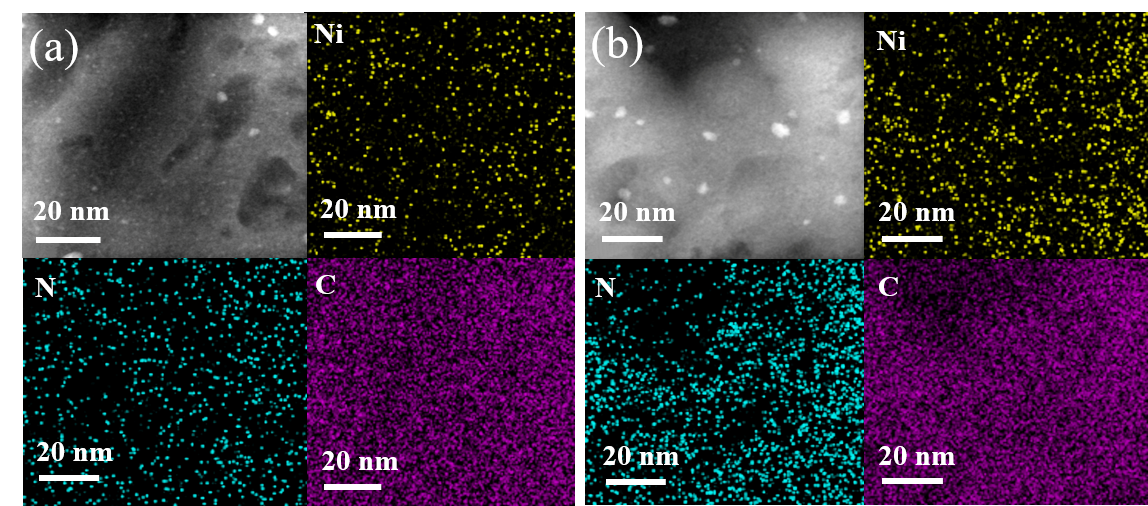


**Figure S7.** a) and b) HR-TEM images (including corresponding EDS mappings of Ni, C and N elements) of Ni/NC and Acid-Ni/NC individually

The energy-dispersive X-ray spectroscopy (EDS) images from HRTEM for Ni/NC and Acid-Ni/NC confirmed that Ni, N, C and O elements were homogeneously distributed.


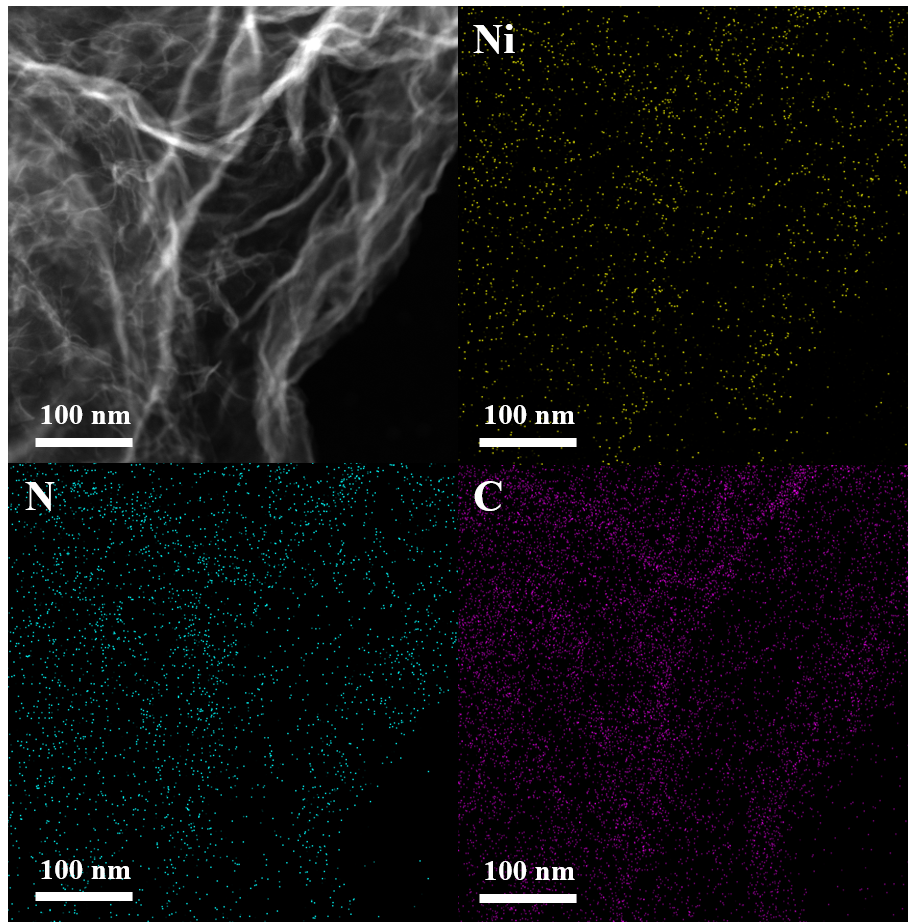


**Figure S8.** HAADF-STEM images of Ar-Ni/NC and corresponding EDS mappings of Ni, C, N and O elements for different sizes.

The energy-dispersive X-ray spectroscopy (EDS) images from HAADF-STEM for Ar-Ni/NC confirmed that Ni, N, C and O elements were homogeneously distributed.


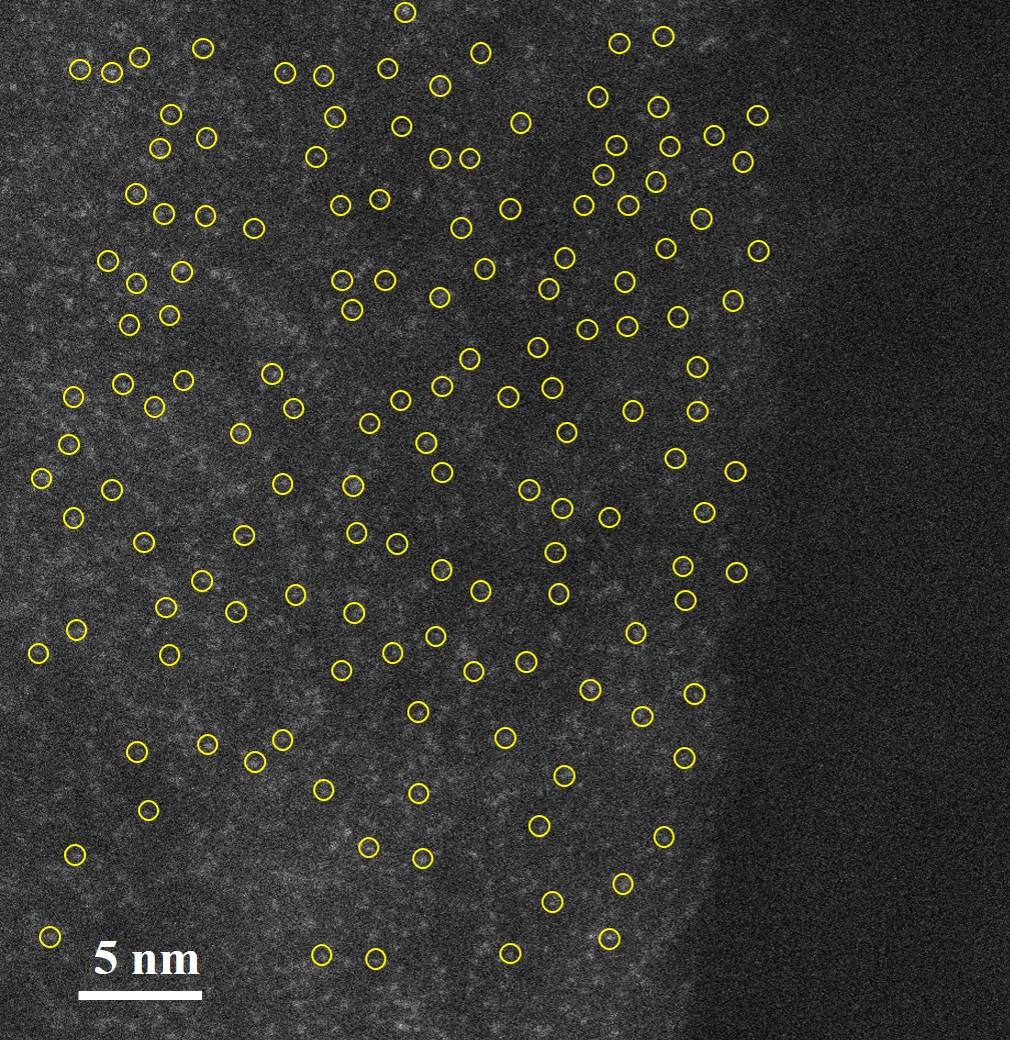


**Figure S9.** AC-HAADF-STEM image of Ar-Ni/NC (the small red circles are Ni single atoms).

The isolated bright spots visible in the figure indicate individual nickel atoms, uniformly dispersed over the carbon matrix. It indicates that the nickel in Ar-Ni/NC exists as single atoms.


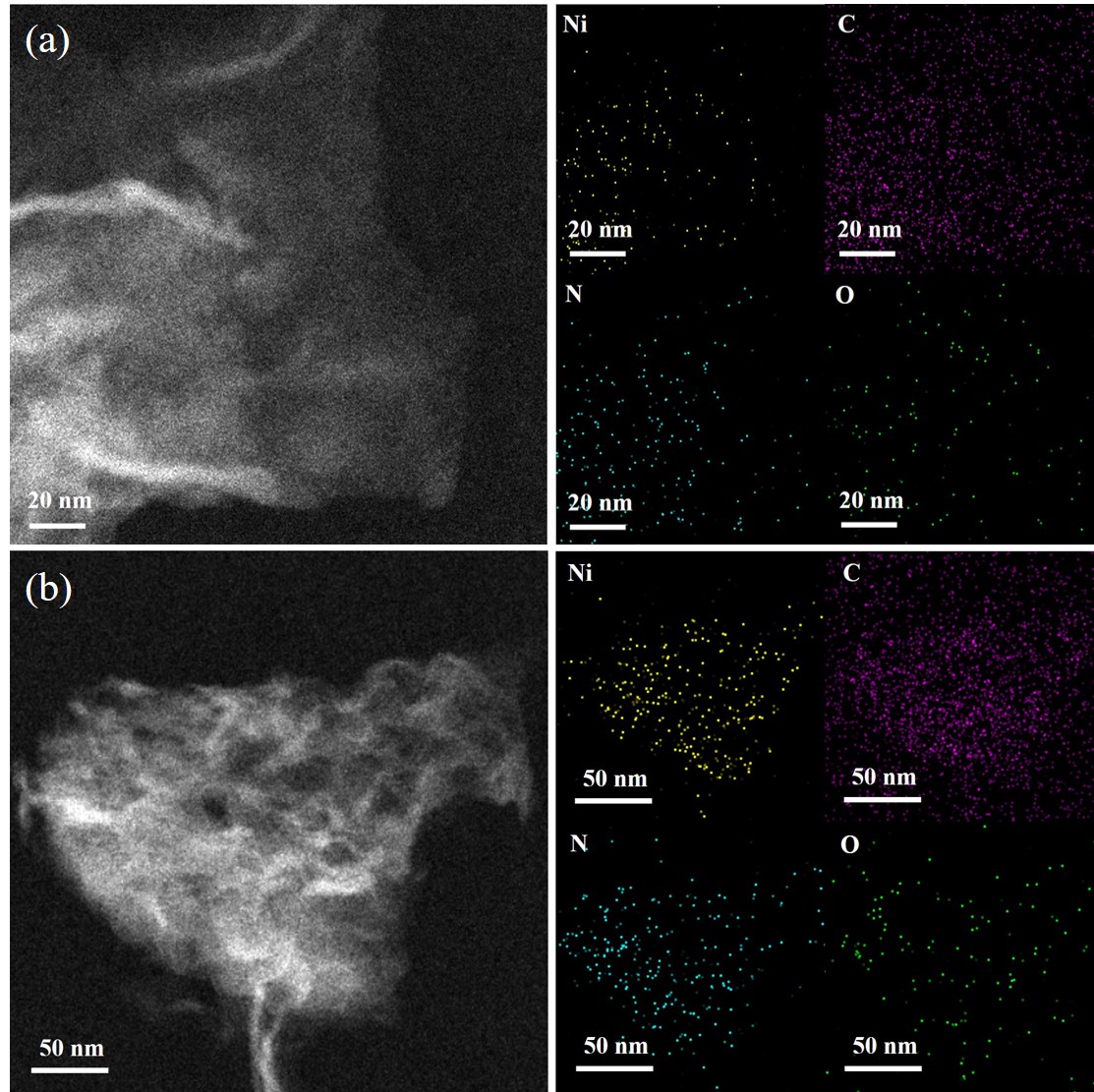


**Figure S10.** AC-HAADF-STEM image of Ar-Ni/NC with different magnifications and corresponding EDS mappings of Ni, C, N and O elements.

From the above spherical aberration electron microscopy, the thin layer structure of Ar-Ni/NC can be clearly seen, and Ni, N and O are uniformly distributed on the carbon matrix.


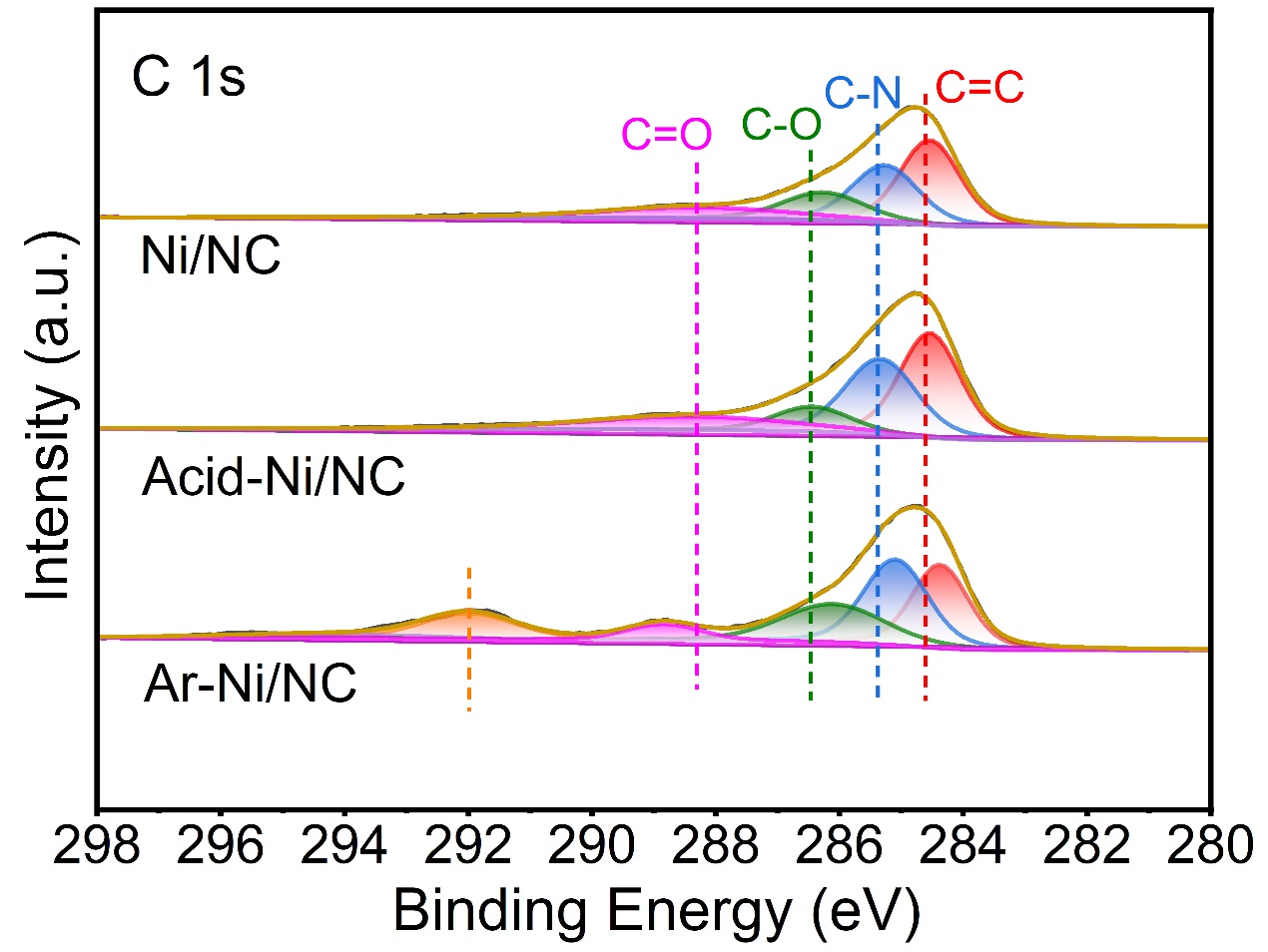


**Figure S11.** C 1s spectra of various Ni/NC catalysts.

The C 1s spectra of as-prepared catalysts were divided into C=C (284.5±0.1 eV), C-N (285.2±0.1 eV), C-O (286.3±0.2 eV) and C=O (288.5±0.2 eV), which indicate that N atoms have been successfully doped into the carbon skeleton. Moreover, the peak at 292 eV in the spectrum is carbon contamination, which is not related to the catalyst in this study.


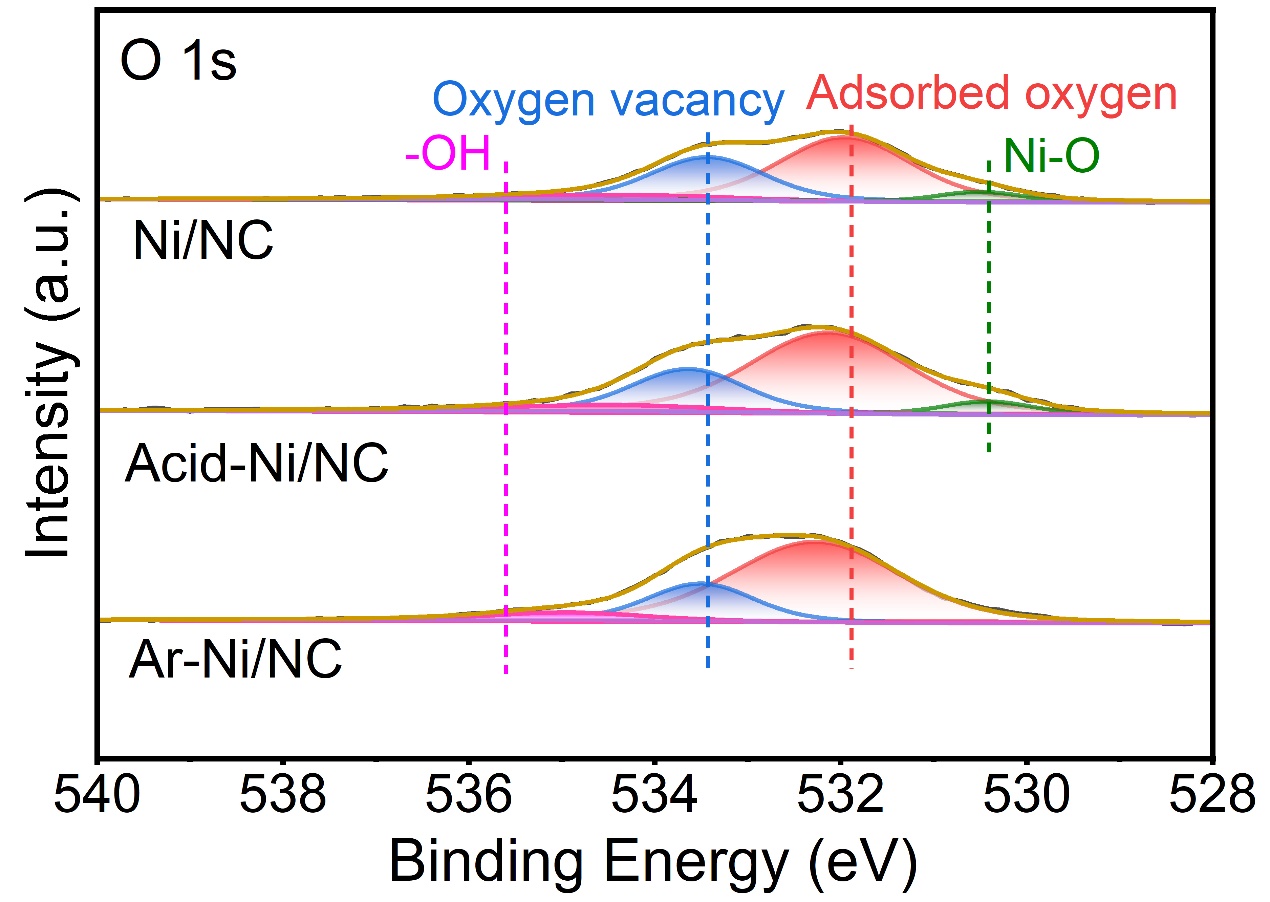


**Figure S12.** O 1s spectra of various Ni/NC catalysts.

For the peak splitting analysis of O 1s spectra, all the prepared catalysts contain the following three peaks: Adsorbed oxygen (~532.1eV), Oxygen vacancy (~533.5eV) and -OH (~534.5eV). Moreover, Ar-Ni/NC doesn’t contain Ni-O bonds (~530.5eV).


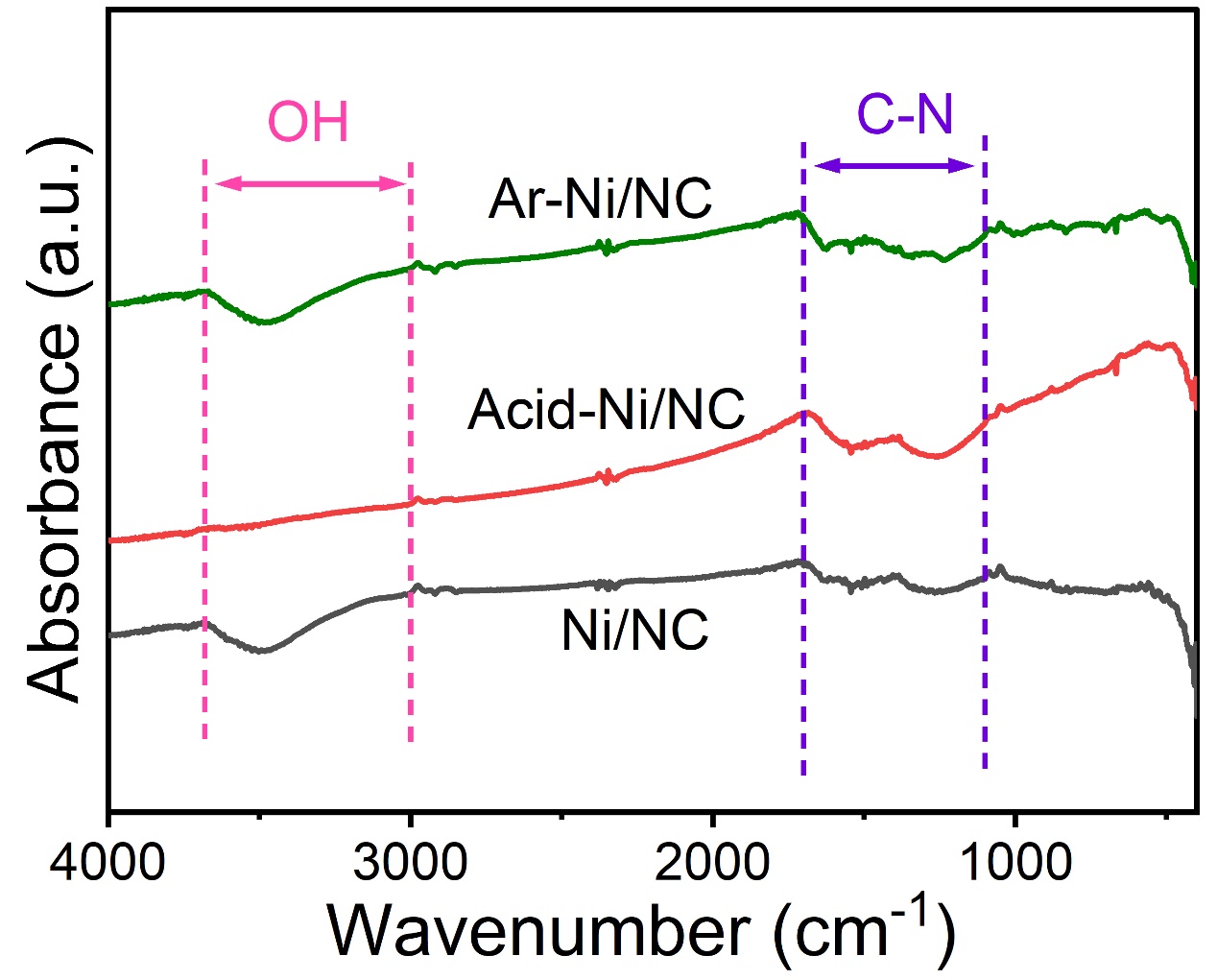


**Figure S13.** FT-IR spectra of various Ni/NC catalysts.

O-H bonds can be attributed to the surface-absorbed H_2_O molecules, and the C-N bonds suggest synergistic effects of N in the carbon network.


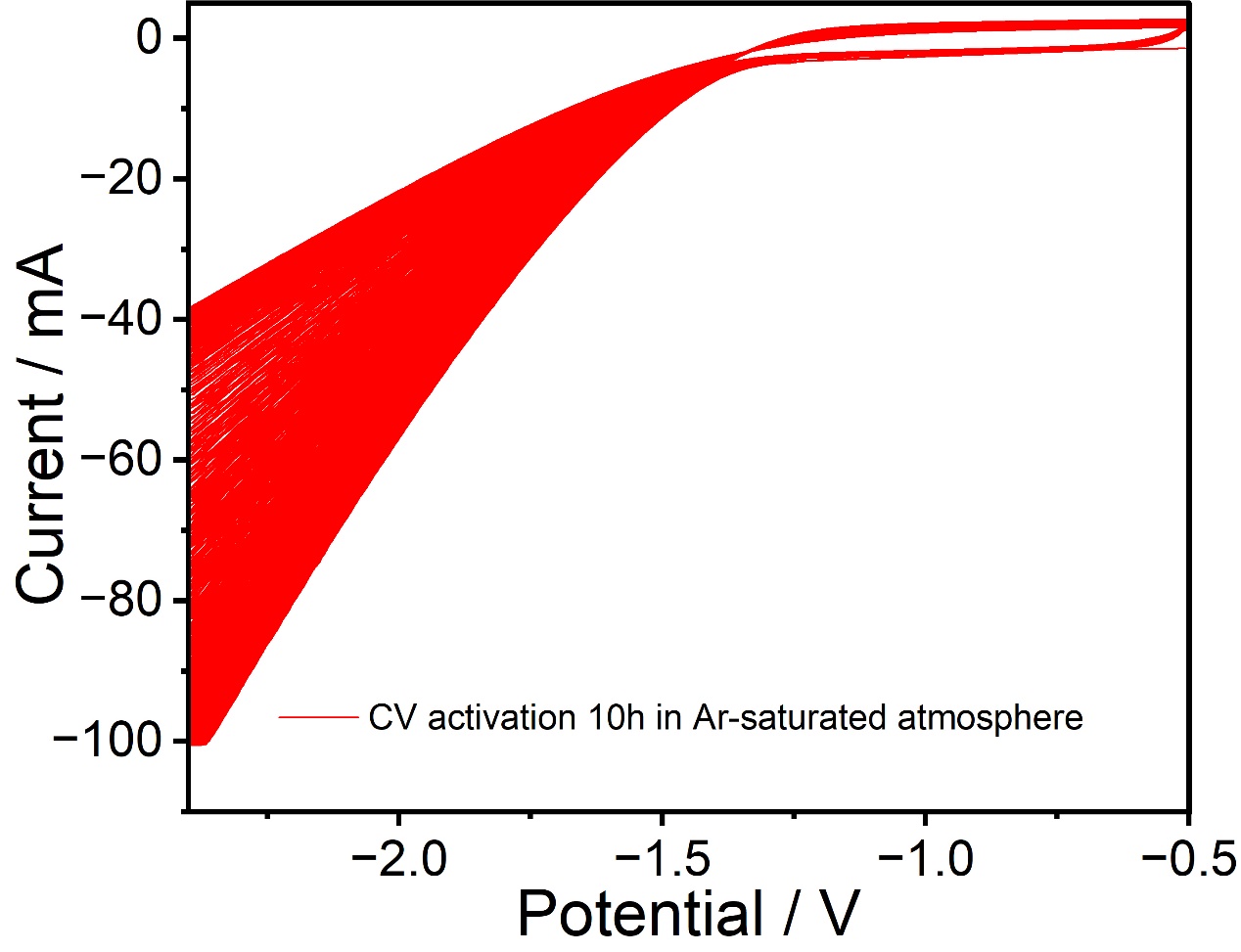


**Figure S14.** Ni/NC in Ar-saturated atmosphere for 10h

The results obtained after electrochemical CO_2_RR activation of the catalyst Ni/NC in the voltage interval -0.5V to -2.4V (vs. SCE) using the CV procedure in Ar atmosphere for 10h.


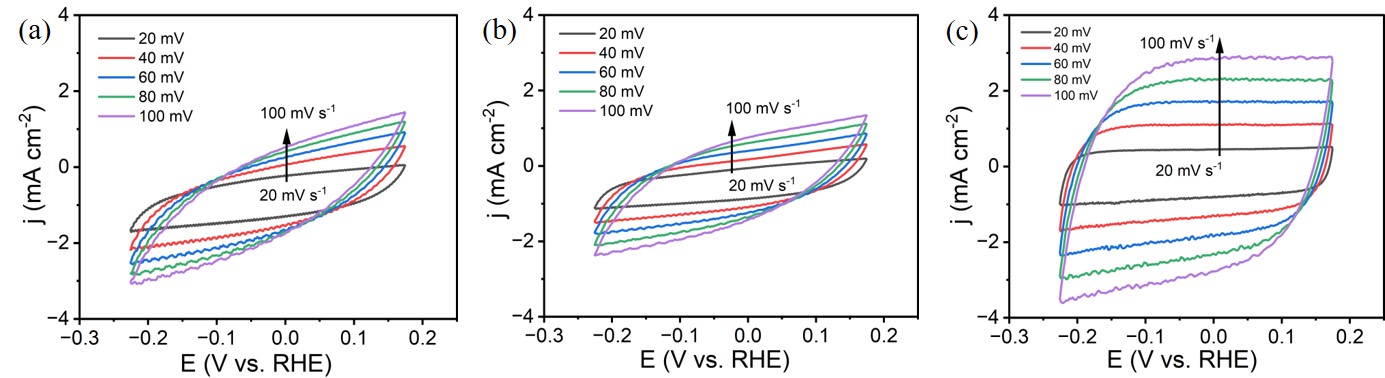


**Figure S15.** a) b) c) Scan rate-dependent CV curves at the nonfaradaic potential region for Ni/NC, Acid-Ni/NC and Ar-Ni/NC.

Test the CV at different scan rates (20, 40, 60, 80 and 100 mV s^-1^) for various catalysts.


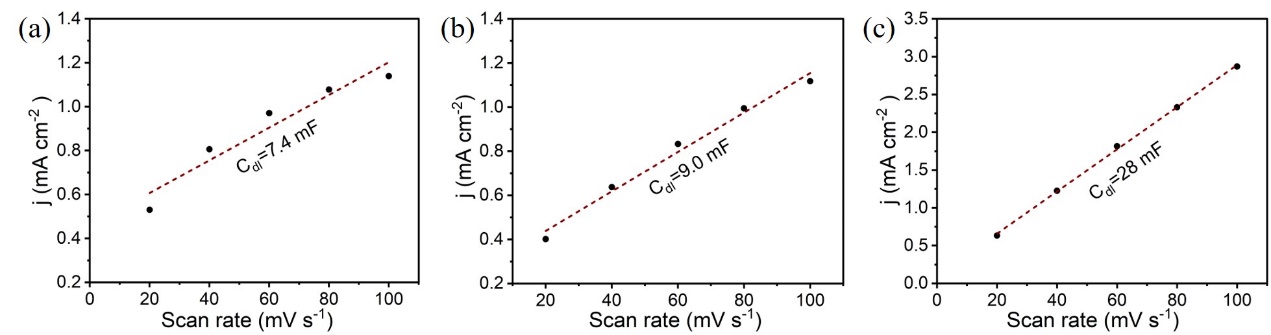


**Figure S16.** a) b) c) electrochemical C_dl_ for Ni/NC, Acid-Ni/NC, and Ar-Ni/NC.

The three diagrams above show the capacitive current density as a function of scan rate for various catalysts.


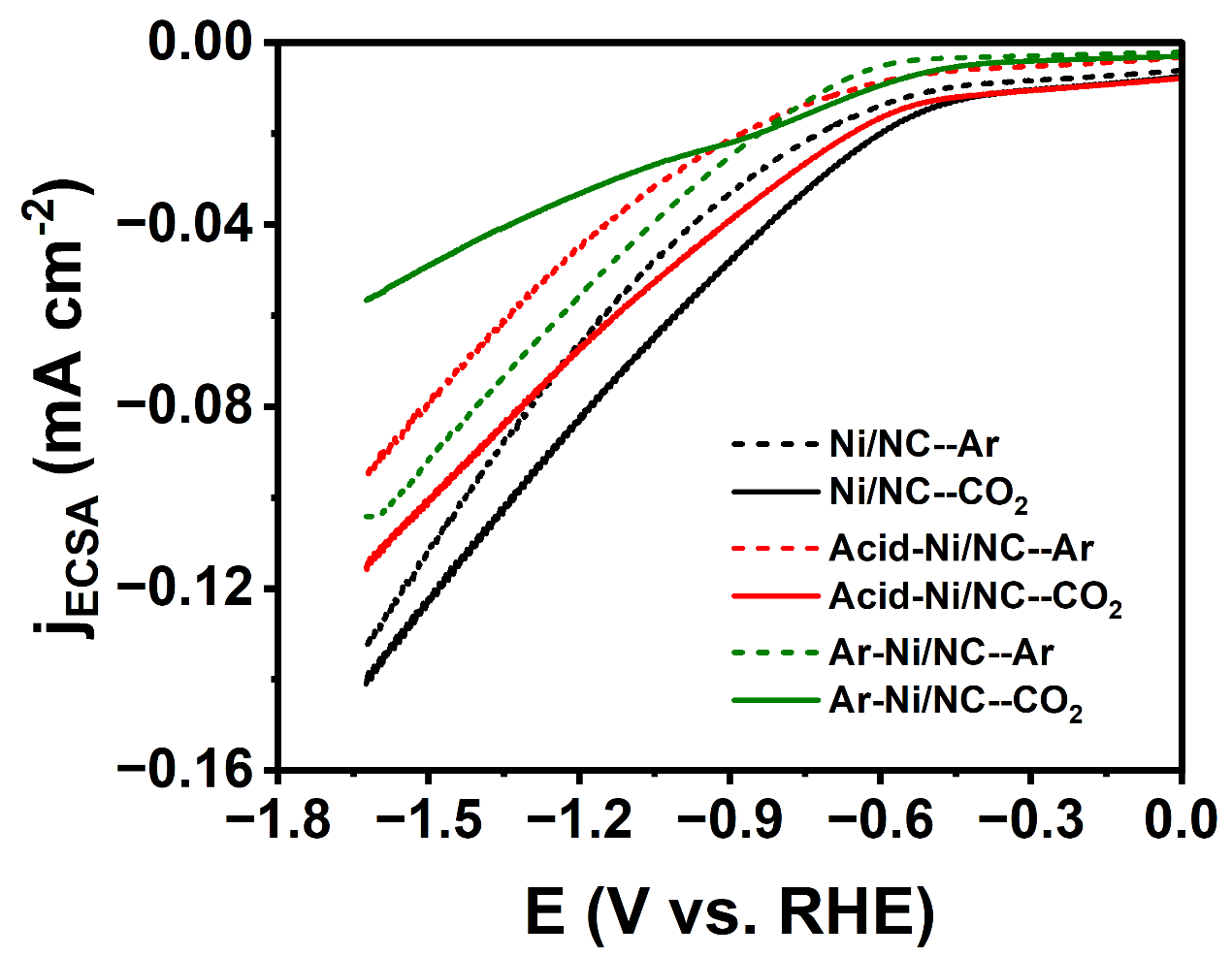


**Figure S17.** j_ECSA_ of various Ni/NC catalysts in CO_2_-saturated 0.1 M potassium bicarbonate (KHCO₃) electrolyte.

In addition, we also normalized the LSV by ECSA. All catalysts showed an overall increase in current density after normalization by ECSA compared to the previous lsv.


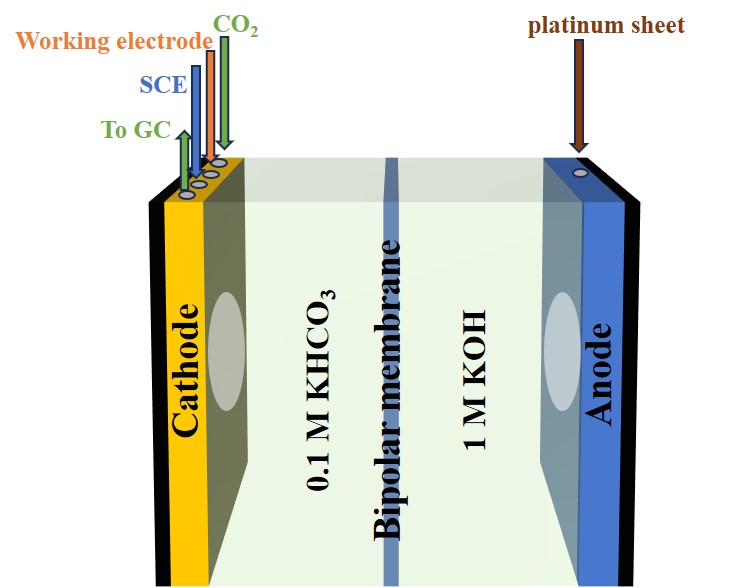


**Figure S18.** Schematic of H-cell reactor used for CO_2_RR.


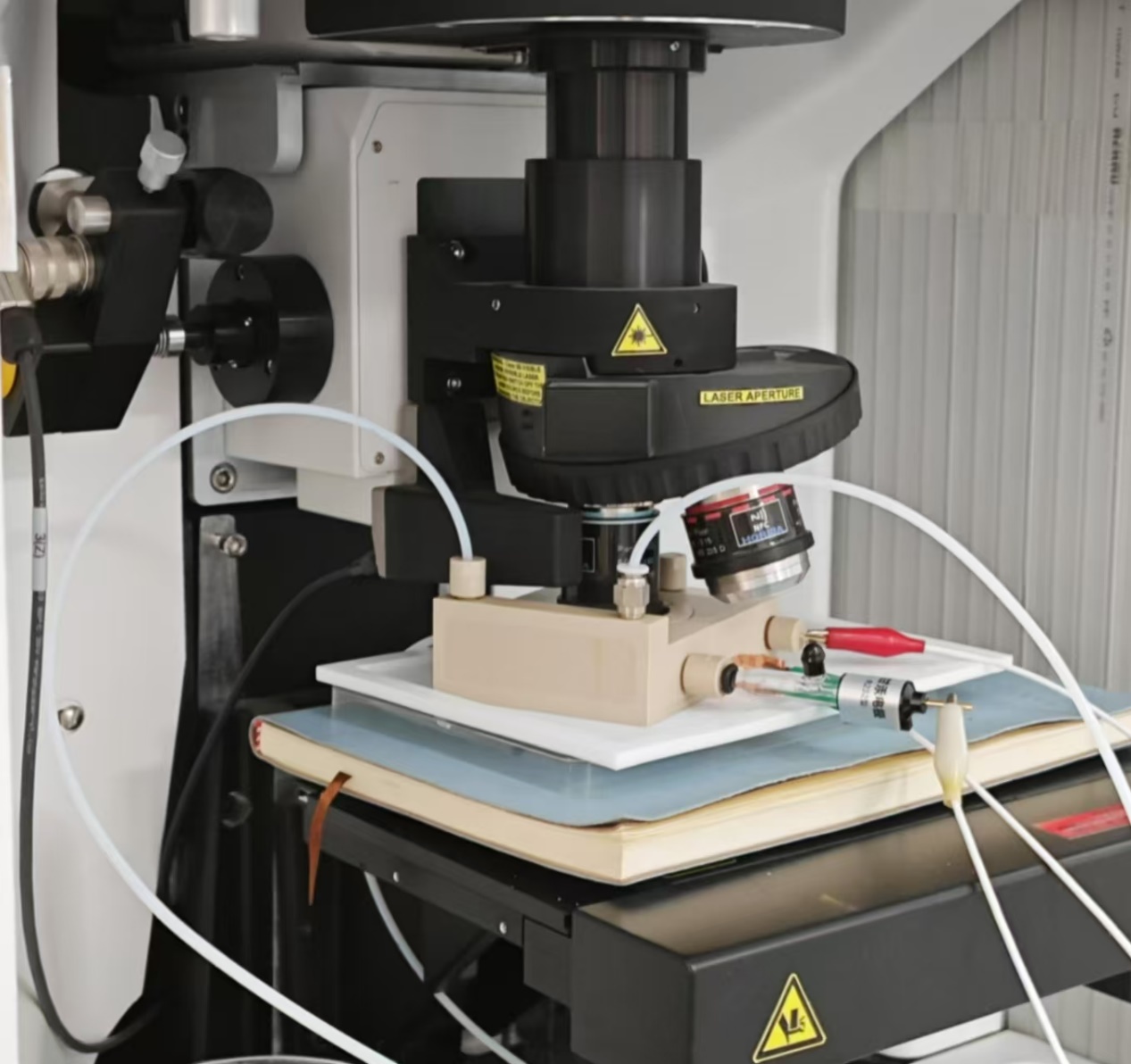


**Figure S19.** In situ Raman measurement setup for CO_2_RR experiments.

The picture shows the device for testing in-situ Raman. In situ Raman spectra were performed using the Labram Soleil Raman spectrometer (HORIBA Jobin Yvon) with the laser of λ = 532 nm and 50x eyepiece.

**Table S1.** Summary of the relative proportions of the three N types (pyridinic, pyrrolic, oxidized) in this word.

| Catalyst | Pyridinic N (%) | Pyrrolic N (%) | Oxidized N (%) |
| --- | --- | --- | --- |
| Ni/NC | 69.60 | 30.40 | 0 |
| Acid-Ni/NC | 60.18 | 29.62 | 10.20 |
| Ar-Ni/NC | 65.74 | 25.40 | 8.85 |

**Table S2.** Summary of the atomic percentage and mass ratio of Ni in SEM catalysts in this word.

| Atomically dispersed metal atom | Catalyst | Metal loading  (at%) | Metal loading  (wt%) |
| --- | --- | --- | --- |
| Ni | Ni/NC | 1.4 | 6.0 |
| Ni | Acid-Ni/NC | 0.4 | 2.0 |
| Ni | Ar-Ni/NC | 0.5 | 2.7 |

**Table S3.** Summary of CO_2_RR to CO on Ni-based catalysts in H-cell.

| Catalyst | Electrolyte | Potential  (V vs. RHE) | FE_CO_ (%) | j_CO_  (mA cm^-2^) | Reference |
| --- | --- | --- | --- | --- | --- |
| Ni/NC | 0.1 M KHCO_3_ | -0.8 | 86.47 | 9.5 | This work |
| Acid-Ni/NC | 0.1 M KHCO_3_ | -0.8 | 91.28 | 9.3 | This work |
| Ar-Ni/NC | 0.1 M KHCO_3_ | -0.8 | 90.48 | 14.7 | This work |
| Ni_0.037_-NG  Ni-N_3_-C  HP-Ni-NC-2  Ni@N-BPC  Ni-N_4_-O/C  Ni SAs | 0.5 M KHCO_3_  0.5 M KHCO_3_  0.5 M KHCO_3_  0.5 M KHCO_3_  0.5 M KHCO_3_  0.5 M KHCO_3_ | -0.8  -0.65  -0.9  -0.9  -0.9  -0.8 | ＞97  95.6  97  98.41  99.2  97 | ＞12  6.64  15  12.58  6  10 | [1]  [2]  [3]  [4]  [5]  [6] |
| Ni-NC@Ni | 0.5 M KHCO_3_ | -0.67 | 87 | 14.8 | [7] |
| Ni SAs/N-C | 0.5 M KHCO_3_ | -0.89 | 70.3 | 10.48 | [8] |
| Ni-SACs | 0.1 M KHCO_3_ | -1.2 | 98.9 | 8 | [9] |
| Ni SAs/N-C | 0.5 M KHCO_3_ | -0.9 | 71.9 | 5.3 | [10] |
| NC-CNT-Ni | 0.1 M KHCO_3_ | -1.0 | 90 | 10 | [11] |
| Ni^2+^@NG | 0.5 M KHCO_3_ | -0.89 | 92 | 12.9 | [12] |

**Reference**

1. S. Liang, Q. Jiang, Q. Wang, Y. Liu, *Adv. Energy Mater*. **2021**, *11*(36).
2. Y. Zhang, L. Jiao, W. Yang, C. Xie, H.L. Jiang, *Angew. Chem., Int. Ed.* **2021**, *60*(14), 7607-7611.
3. C. Zhou, R. Zhang, Y. Rong, Y. Yang, X. Jiang, *ACS Appl. Mater. Interfaces* **2023**, *15*(36), 42585-42593.
4. S. Zhao, K. Zhang, L. Wu, X. Du, S. Sun, Y. Wang, J. Li, Z. Zhang, *Energy & Fuels* **2024**, *38*(13), 11909-11917.
5. X. Wang, Y. Wang, X. Sang, W. Zheng, S. Zhang, L. Shuai, B. Yang, Z. Li, J. Chen, L. Lei, N.M. Adli, M.K.H. Leung, M. Qiu, G. Wu, Y. Hou, *Angew. Chem., Int. Ed*. **2021**, *60*(8), 4192-4198.
6. Z. Li, D. He, X. Yan, S. Dai, S. Younan, Z. Ke, X. Pan, X. Xiao, H. Wu, J. Gu, *Angew. Chem., Int. Ed*. **2020**, *59*(42),18572-18577.
7. Y. He, Y. Li, J. Zhang, S. Wang, D. Huang, G. Yang, X. Yi, H. Lin, X. Han, W. Hu, Y. Deng, J. Ye, *Nano Energy* **2020**, *77*.
8. C. Zhao, X. Dai, T. Yao, W. Chen, X. Wang, J. Wang, J. Yang, S. Wei, Y. Wu, Y. Li, *J. Am. Chem. Soc*. **2017**, *139*(24), 8078-8081.
9. H. Yang, L. Shang, Q. Zhang, R. Shi, G.I.N. Waterhouse, L. Gu, T. Zhang, *Nat. Commun*. **2019**, *10*(1).
10. J. Leverett, J.A. Yuwono, P. Kumar, T. Tran-Phu, J. Qu, J. Cairney, X. Wang, A.N. Simonov, R.K. Hocking, B. Johannessen, L. Dai, R. Daiyan, R. Amal, *ACS Energy Lett*. **2022**, *7*(3), 920-928.
11. Q. Fan, P. Hou, C. Choi, T.S. Wu, S. Hong, F. Li, Y.L. Soo, P. Kang, Y. Jung, Z. Sun, *Adv. Energy Mater*. **2019**, *10*(5).
12. W. Bi, X. Li, R. You, M. Chen, R. Yuan, W. Huang, X. Wu, W. Chu, C. Wu, Y. Xie, *Adv Mater*. **2018**, *30*(18).
